# Mechanisms controlling replication fork stalling and collapse at topoisomerase 1 cleavage complexes

**DOI:** 10.1016/j.molcel.2024.08.004

**Published:** 2024-09-04

**Authors:** Rose Westhorpe, Johann J. Roske, Joseph T.P. Yeeles

**Affiliations:** 1 Protein and Nucleic Acid Chemistry Division, Medical Research Council, Laboratory of Molecular Biology, Cambridge CB2 0QH, UK

## Abstract

Topoisomerase 1 cleavage complexes (Top1-ccs) comprise a DNA-protein crosslink and a single-stranded DNA break that can significantly impact the DNA replication machinery (replisome). Consequently, inhibitors that trap Top1-ccs are used extensively in research and clinical settings to generate DNA replication stress, yet how the replisome responds upon collision with a Top1-cc remains obscure. By reconstituting collisions between budding yeast replisomes, assembled from purified proteins, and site-specific Top1-ccs, we have uncovered mechanisms underlying replication fork stalling and collapse. We find that stalled replication forks are surprisingly stable and that their stability is influenced by the template strand that Top1 is crosslinked to, the fork protection complex proteins Tof1-Csm3 (human TIMELESS-TIPIN), and the convergence of replication forks. Moreover, nascent-strand mapping and cryoelectron microscopy (cryo-EM) of stalled forks establishes replisome remodeling as a key factor in the initial response to Top1-ccs. These findings have important implications for the use of Top1 inhibitors in research and in the clinic.

## Graphical abstract

**Graphic abstract:**
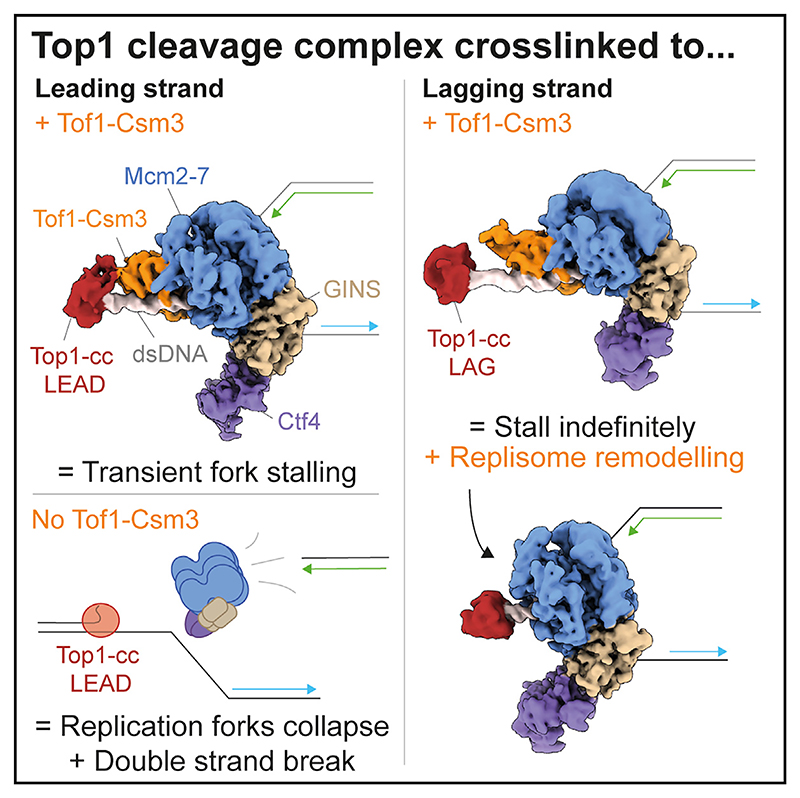

## Introduction

Accurate and efficient chromosome replication is critical for faithful genome inheritance. In addition to the inherent complexity and vast scale of this process, the DNA replication machinery (replisome) frequently encounters obstacles to its progression that can lead to DNA damage and mutagenesis.^1,2^ DNA-protein crosslinks (DPCs) are a class of DNA lesion that can be generated by exposure to exogenous agents, such as ionizing radiation and platinum-based drugs, but also arise endogenously when DNA-modifying enzymes are trapped on their substrate.^3,4^ DPCs present a physical barrier to replisome progression, preventing DNA unwinding by the Cdc45-MCM-GINS (CMG) replicative helicase and inhibiting DNA polymerases.^5–10^

A major form of DPC is generated by the enzymatic activity of topoisomerase 1 (Top1). Top1 assists replisome progression by resolving torsional stress in the DNA that accumulates ahead of the replication fork.^11–14^ This is achieved via formation of transient single-stranded breaks, or nicks, when a tyrosine residue in the active site becomes crosslinked to the DNA back-bone.^15–18^ The resulting intermediate, termed a Top1 cleavage complex (Top1-cc), allows for relaxation of the DNA through a controlled rotation mechanism.^17,19,20^ These intermediates are normally quickly reversed but can be stabilized if Top1-mediated DNA re-joining is inhibited.^21^ Genetic studies in budding yeast indicate that this is a frequent occurrence because removal of Wss1 and Tdp1—which both have roles in Top1-cc repair—is highly toxic, demonstrating that endogenous levels of Top1-ccs must be continually resolved.^22^

The plant alkaloid camptothecin (CPT) is a potent Top1 inhibitor. It intercalates at the site of Top1-induced DNA cleavage, preventing re-ligation of the break and stabilizing the Top1-cc.^23–26^ CPT treatment leads to the accumulation of single-stranded DNA (ssDNA) and double-stranded DNA (dsDNA) breaks in cells, DNA damage checkpoint activation, and cytotoxicity.^27^ This cytotoxicity can be suppressed by treatment of cells with aphidicolin or hydroxyurea, which slow down DNA replication, or by inhibiting entry into S phase, suggesting that the primary mechanism for Top1-cc-induced cell death is collision with replication forks and the ensuing DNA damage.^24,28,29^ The sensitivity of proliferating cells to Top1-ccs has led to the use of CPT derivatives as anticancer drugs.^27,30^

Evidence from cellular studies suggests that collisions between replication forks and Top1-ccs can have a variety of different outcomes. CPT treatment has been shown to generate single-ended dsDNA breaks on the leading strand in a replication-dependent manner,^31^ presumably due to replication fork collapse at the Top1-cc. However, following low doses of CPT, replication forks were found to undergo fork reversal,^32^ indicating that replisomes can stall at Top1-ccs without the replication fork collapsing. Consistent with these results, dsDNA breaks can be suppressed if proteolytic processing of the Top1-cc is inhibited.^33,34^ Thus, the interplay between replication fork stalling and collapse at a Top1-cc is complex and the underlying mechanisms are yet to be fully resolved, at least in part because the initial response of the replisome to a Top1-cc is unknown. For example, it is yet to be established how the template strand to which the Top1-cc is linked influences the collision, nor is it clear how different components of the core replisome modulate its behavior. One such component is the fork protection complex (FPC). The FPC, which contains the Tof1-Csm3 heterodimer (human TIMELESS-TIPIN), sits on the leading edge of the CMG and has multiple functions during replisome progression, including to promote replication fork pausing at certain proteinaceous barriers.^35–38^ Budding yeast cells lacking Tof1 or Csm3^39^ and avian cells lacking TIPIN^40^ are hypersensitive to CPT, and human cells depleted of TIMELESS or TIPIN experience high chromosomal breakage following CPT treatment.^41^ Together, these data indicate a key role for the FPC in the response to Top1-ccs; however, the mechanism through which it functions remains elusive.

How the replisome initially responds when it collides with a Top1-cc is likely to be a key factor in determining how the lesion is bypassed and/or repaired. Because many different DNA repair factors are deployed in response to CPT treatment and check-point proteins are activated, it is challenging to deconvolute the earliest responses of the replisome to a Top1-cc from down-stream processing events using cell-based approaches. To overcome these challenges, we have reconstituted collisions between replisomes assembled from purified *Saccharomyces cerevisiae* (*S. cerevisiae*) proteins and site-specific Top1-ccs. Using a combination of origin-specific DNA replication assays and cryoelectron microscopy (cryo-EM) analysis of stalled replication forks, our work reveals how the replisome initially responds to Top1-ccs, the architecture of stalled replication forks, and mechanisms that influence the balance between replication fork stalling and collapse.

## Results

### Generation of site-specific Top1-ccs *in vitro*

To generate DNA templates with site-specific and stable Top1-ccs, we adopted a similar strategy to that used previously to obtain crystal structures of Top1-cc DNA complexes^18^ (Figure S1). A single 5′ bridging phosphorothioate (OPS) backbone modification was inserted within a 25-nucleotide (nt) sequence that has previously been reported to exhibit preferential Top1 binding and cleavage^42^ (Figure S1A). Cleavage of the DNA backbone by Top1 at the OPS modification generates a free 5′ sulfhydryl that is not competent for re-ligation, thereby forming long-lived Top1-cc intermediates (Figure S1B).^43^ Stable, site-specific Top1-ccs should result in persistent ssDNA nicks at the site of the OPS modification. Accordingly, incubation of Top1 with a 9.7-kb linear 5′ end-labeled DNA containing the OPS modification generated a 5.2-kb cleavage product in a denaturing agarose gel, the size of which corresponds to the distance from the 5′ end label to the OPS modification (Figures S1C and S1D). Cleavage was highly efficient, consistent with previous studies,^43^ and was dependent on both the OPS modification and Top1, indicating that under our reaction conditions the OPS containing DNA is stable and the DNA sequence alone is insufficient to form long-lived Top1-ccs.

### Top1-ccs are a potent block to leading-strand DNA replication

**Figure 1:**
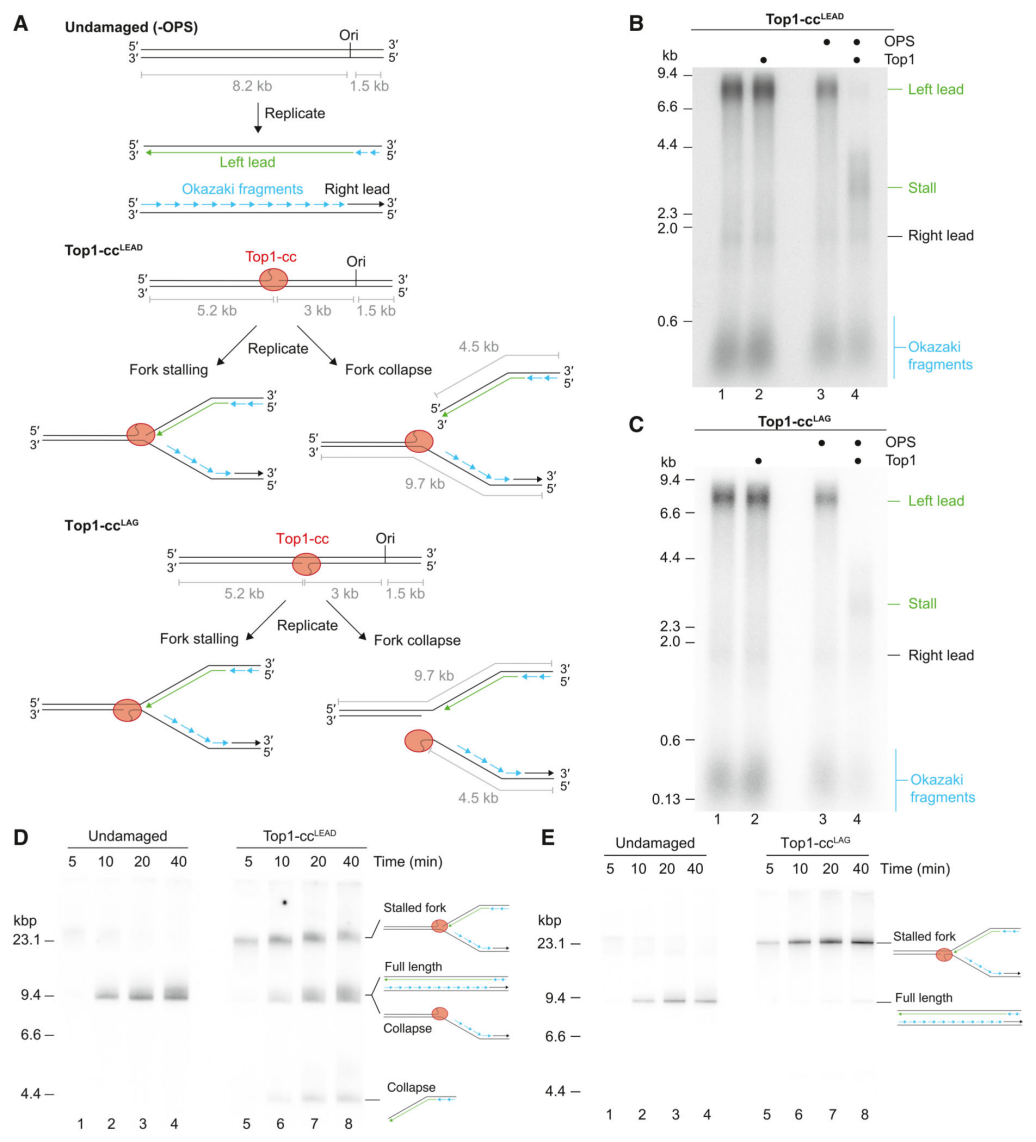
The orientation of the Top1-cc strongly influences replication fork stalling and collapse (A) Schematic of undamaged (–OPS) and Top1-cc (OPS containing) linear DNA replication templates used in this study. For each template, the possible outcomes and resulting product sizes are indicated. In all figures, Ori marks the position of the replication origin ARS306 on the template. Nascent leading strands from leftward-moving replication forks are shown in green and nascent lagging strands (Okazaki fragments) are shown in blue. (B and C) Denaturing agarose gel analysis of replication products generated from the templates illustrated in (A). Reactions were quenched at 15 min. (D and E) Native agarose gel analysis of replication products from time course experiments on the templates illustrated in (A).

With a system in place to efficiently generate site-specific Top1-ccs, we sought to investigate how the core replisome responds to these lesions using an *in vitro* DNA replication system. In this system, replisomes that perform complete leading- and lagging-strand DNA replication at the *in vivo* rate are assembled at replication origins from purified *S. cerevisiae* proteins.^44,45^ Nascent replication products are radiolabeled during the reaction by inclusion of [α-32P]-dCTP or [α-32P]-dATP and are visualized by autoradiography after separation through agarose gels. By using linear DNA templates with the origin positioned off center, the progression of unidirectional populations of replisomes can be monitored.^46^ To examine the impact of leading-strand Top1-cc lesions (Top1-cc^LEAD^) and lagging-strand lesions (Top1-cc^LAG^), we constructed 9.7-kb linear templates with the OPS modification positioned ~3 kb to the left of the replication origin (ARS306) in either the leading- or lagging-strand template (Figure 1A). In the absence of Top1 or the OPS modification, ~8.2-kb leftward-moving leading strands (left lead) and ~1.5-kb right-ward-moving leading strands (right lead) were synthesized, in addition to Okazaki fragments that remained unligated due to omission of Fen1 and ligase that are required for Okazaki maturation^47^ (Figures 1B and 1C). In reactions containing OPS templates and Top1, full-length 8.2-kb left leading strands were almost completely absent. Instead, for both Top1-cc^LEAD^ (Figure 1B, lane 4) and Top1-cc^LAG^ (Figure 1C, lane 4), the majority of left-leading-strand products migrated as a broad smear centered on ~3 kb. This size distribution is consistent with leading-strand synthesis being arrested in the vicinity of the Top1-cc because leading-strand synthesis is initiated over a broad region, which generates an inherent heterogeneity in leading-strand product length.^46^ These results demonstrate that a Top1-cc is a potent block to leading-strand DNA replication, irrespective of which template strand the topoisomerase is covalently attached to, and that a Top1-cc was formed on most replicated DNA templates.

### The orientation of the Top1-cc strongly influences replication fork stalling and collapse

Crystal structures of Top1-ccs showed that the topoisomerase remains in contact with both strands of the DNA duplex, with the nick buried inside the protein (Figure S1B).^17,18,25,26^ Therefore, if the replisome is unable to displace the topoisomerase from the template, 3-kb leading strands could result from prolonged replication fork stalling (Figure 1A). Alternatively, at Top1-cc^LEAD^, replication forks would collapse upon displacement of Top1 and translocation of the CMG helicase off the template at the nick generated by Top1. Similarly, at Top1-cc^LAG^, 3-kb leading strands would be observed if replication forks collapsed due to displacement of Top1 from the template and subsequent CMG translocation to the nick.^48^ To distinguish between these outcomes, we performed time-course experiments and analyzed the products on native agarose gels that preserve the structures of replication intermediates and stalled replication forks.^46^ Whereas the majority of products from undamaged templates migrated at the position of full length after 10 min, replication of the Top1-cc^LEAD^ template generated a slowly migrating species indicative of replication fork stalling^46^ (Figure 1D, lanes 5–8, stalled fork). As the reaction progressed, stalled forks became less abundant and products migrating in the position of full length (9.7 kb) accumulated, as did a shorter product of ~ 4.5 kb, indicating that replication forks were collapsing at the Top1-cc^LEAD^ nick (Figures 1A and 1D). Two-dimensional (2D) gel analysis confirmed this conclusion: the 9.7-kb product was comprised mostly of Okazaki fragments and 1.5-kb right leading strands, whereas the 4.5-kb product contained predominantly arrested ~3-kb leading strands (Figures S2A and S2B). Thus, the initial response of the core eukaryotic replisome is to stall at Top1-cc^LEAD^, often for several minutes, before most replication forks eventually collapse to generate single-ended dsDNA breaks in this unidirectional replication system.

Native and 2D agarose gel analysis of replisome collisions with Top1-cc^LAG^ revealed a strikingly different outcome (Figures 1E and S2C). Stalled replication forks accumulated throughout the reaction time course and, even after 40 min, products indicative of replication fork collapse were barely detectable. These data reveal that replisomes encountering Top1-cc^LAG^ form highly stable stalled replication forks and that the orientation of the Top1-cc is a key factor in determining the initial outcome of a collision with the replisome.

### The disposition of stalled replication forks is influenced by Top1-cc orientation

**Figure 2:**
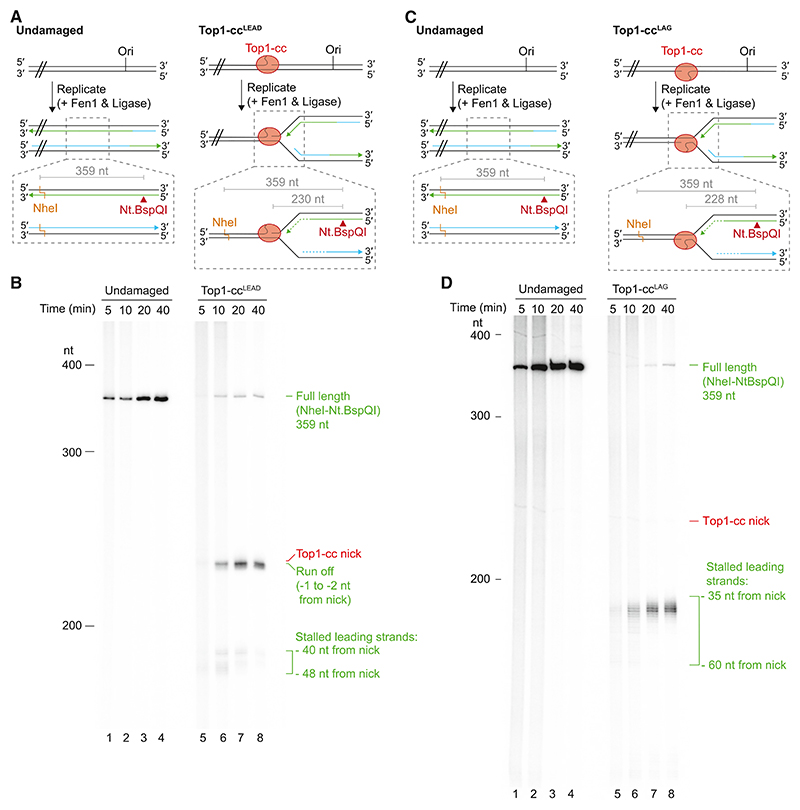
The disposition of stalled replication forks is influenced by Top1-cc orientation (A) Schematic of the post-reaction processing strategy used to liberate nascent leading strands from reactions on undamaged and Top1-cc^LEAD^ templates for denaturing polyacrylamide gel analysis. Nascent leading strands are shown in green and matured lagging strands are shown in blue. Expected product sizes, indicated in gray, were calculated from the Top1-cc nick site to the respective site of strand cleavage for restriction enzymes, or between two restriction enzyme sites. For clarity, the region of the template downstream of the dashed lines (//) is not shown. (B) Denaturing polyacrylamide gel analysis of replication products from a time course experiment on undamaged and Top1-cc^LEAD^ templates, processed as indicated in (A). (C) As in (A) for replication reactions performed on undamaged or Top1-cc^LAG^ templates. (D) Denaturing polyacrylamide gel analysis of replication products from a time course experiment on undamaged and Top1-cc^LAG^ templates, processed as indicated in (C).

To gain insight into the disposition of stalled and collapsed replication forks at Top1-cc^LEAD^, we mapped nascent leading-strand products using denaturing urea/polyacrylamide gels. After replication, products were digested with the nicking enzyme Nt.BspQI, which cleaves nascent leading strands 230 nt upstream of the Top1-cc nick (Figure 2A). To visualize nascent leading strands from templates lacking a Top1-cc, products were also digested with NheI, which digests dsDNA downstream of the Top1-cc, such that a double digest will generate a 359-nt product if leading-strand synthesis is not arrested (Figure 2A). Nascent lagging strands are not cut by Nt.BspQI and therefore remain too long to be resolved. To aid the visualization of leading-strand cleavage products, Fen1 and ligase were included in the reactions to promote Okazaki fragment maturation because unligated Okazaki fragments are ~200–600 nt in length (Figures 1B and 1C) and will therefore migrate in the same position as cleavage products. Treatment of replication products from an undamaged template with Nt.BspQI and NheI generated a single 359-nt product (Figures 2B, S3A, and S3B). Analysis of leading strands from the Top1-cc^LEAD^ template revealed three sets of products at the 10-min time point: a faint 359-nt product, likely resulting from a small fraction of the template lacking a Top1-cc; an ~230-nt product (runoff), indicative of leading-strand extension to the Top1-cc nick after fork collapse; and a cluster of products ~180–190 nt in length (stall) that represent arrested leading strands at stalled replication forks. As the reaction progressed, stall products were diminished and runoff became more prominent, confirming that stalled replication forks were collapsing to generate blunt, or nearly blunt, dsDNA breaks. Moreover, failure to detect products migrating between stall and runoff indicates that leading strands were rapidly extended to the Top1-cc nick upon fork collapse. The distribution of stall products demonstrates that leading-strand synthesis was arrested ~40–50 nt upstream of the Top1-cc nick. In *Xenopus* egg extracts, leading-strand synthesis initially stops ~20 nt upstream of an interstrand crosslink due to the footprint of the CMG helicase.^8^ Therefore, because crystal structures of Top1-ccs indicate that ~10 bp of dsDNA are obscured between the nick and the replisome-facing edge of the topoisomerase (Figure S1B), the observed stalling of leading-strand synthesis ~ 40–50 nt from the Top1-cc nick indicates that additional replisome components might influence fork stalling at Top1-cc^LEAD^.

Analysis of nascent leading strands at Top1-cc^LAG^ revealed that, after 10 min, stall products were distributed across a broad zone ~35–60 nt from the Top1-cc nick (Figure 2D). As the reaction progressed, the distribution of stall products became tighter but leading-strand synthesis did not advance beyond the −35 position, even after 40 min (Figures 2D, S3C, and S3D). These findings are consistent with the native gel analysis (Figure 1E) showing that replication forks do not readily collapse at Top1-cc^LAG^ in this system.

### Fork convergence at Top1-ccs can trigger double fork collapse

**Figure 3:**
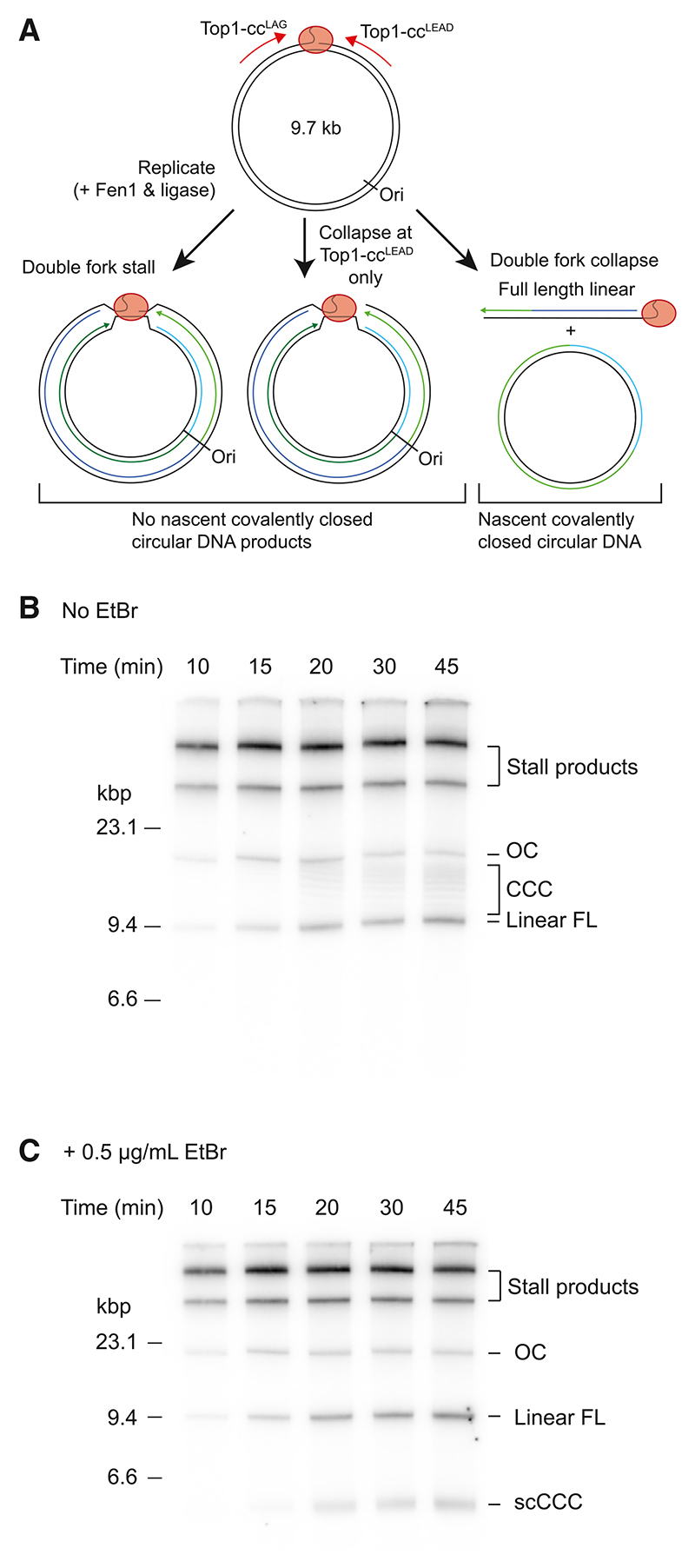
Fork convergence at Top1-ccs leads to double fork collapse (A) Schematic of potential outcomes resulting from replication of a 9.7-kb circular template containing a single Top1-cc, in the presence of Fen1 and ligase. Nascent leading strands are indicated in green shades and matured lagging strands are indicated in blue shades. (B and C) Native agarose gel analysis in the absence (B) or presence (C) of ethidium bromide (EtBr) of products generated from replication reactions as indicated in (A). OC, open circular; CCC, covalently closed circles; linear FL, full length; scCCC, supercoiled covalently closed circles.

Due to the surprising stability of replication forks stalled at Top1-ccs, we reasoned that replisomes from neighboring origins might converge upon a Top1-cc. To model this scenario, we performed replication reactions on 9.7-kb circular DNA templates containing a single Top1-cc (Figure 3A). As the Top1-cc is positioned off center with respect to the origin, anticlockwise moving replication forks—which will encounter the Top1-cc in the leading-strand template—will more frequently encounter the lesion first (Figure 3A). If both replication forks stall and do not collapse, structures resembling late replication intermediates will be generated that migrate slowly in native agarose gels. If only the replication forks that encounter Top1-cc^LEAD^ collapse, both daughter molecules will remain associated via a short unreplicated region and should also migrate slowly in native agarose gels. In contrast, if both replication forks collapse, closed circular dsDNA products should be generated upon complete ligation of one daughter strand as well as an ~9.7-kb linear product with the Top1-cc attached at one end (Figure 3A, double fork collapse).

Replication of a circular Top1-cc template in the presence of Fen1 and ligase generated a range of different products in a native agarose gel, including slowly migrating bands indicative of double stall and single fork collapse events (stall products), full-length linear products, and a product of the expected size for relaxed/open circular dsDNA (Figure 3B). Additionally, a faint ladder of bands was apparent that migrated below the relaxed/open circular products, which we hypothesized might represent closed circular dsDNA products in different topological states. If this assignment is correct, these products should become supercoiled in the presence of ethidium bromide. Accordingly, Figure 3C shows that, in the presence of ethidium bromide, the ladder of bands was no longer visible and a faster-migrating species accumulated across the time course, demonstrating that closed circular dsDNA products were generated, presumably following double replication fork collapse. Migration of the relaxed/open circular population was not altered by ethidium bromide, indicating that these products contained nicks or gaps. Such products could result either from incomplete Okazaki fragment maturation of circular products from double collapse events or from post-replicative collapse of stalled replication forks during sample processing.

To further evaluate the products of double fork collapse events, reaction products from circular templates were digested with SmaI, which cuts adjacent to the origin, before denaturing gel analysis. Full-length nascent ssDNA products should only be observed after SmaI digestion if closed circular products were synthesized, while products from replication forks arrested at the Top1-cc will be digested into distinct ~3-kb and ~6.7-kb products (Figure S4A). After SmaI digestion, full-length ssDNA products accumulated across a time course with similar kinetics to the closed circular dsDNA products (compare Figures 3C and S4B). Moreover, these products were dependent on Fen1 and ligase (Figure S4C). Collectively, these data reveal that both replication forks can collapse when replisomes converge on a Top1-cc, which generates a fully replicated daughter and a daughter with a two-ended dsDNA break. The observation of 3474 Molecular Cell *84*, 3469–3481, September 19, 2024 double fork collapse upon fork convergence, together with the lack of fork collapse at Top1-cc^LAG^ in unidirectional experiments (Figures 1E and 2D), indicates that collision of the replisome on the Top1-cc^LEAD^ side of the lesion, and likely its subsequent collapse, destabilizes the Top1-cc such that it can be displaced by the converging replisome.

### The FPC is essential for pausing at Top1-cc^LEAD^

**Figure 4:**
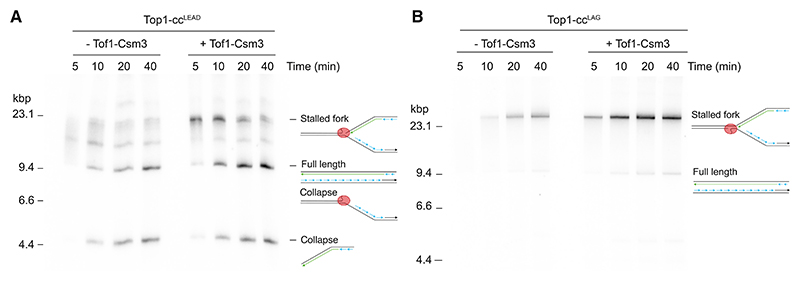
The fork protection complex is essential for pausing at Top1-cc^LEAD^ (A and B) Native agarose gel analysis of replication products generated in time course reactions on linear Top1-cc^LEAD^ (A) and Top1-cc^LAG^ (B) templates, in the presence or absence of Tof1-Csm3.

The FPC proteins Tof1 and Csm3—which promote cellular tolerance of CPT^39,40^—are positioned on the leading edge of the CMG replicative helicase^35^ and are therefore likely one of the first replisome components to encounter an obstacle ahead of the replication fork. We therefore reasoned that Tof1-Csm3 might modulate the behavior of the replisome when it collides with a Top1-cc. To test this idea, we performed time-course experiments in the absence or presence of Tof1-Csm3 and analyzed the products in native agarose gels (Figure 4). Whereas prolonged replication fork stalling was again observed on the Top1-cc^LEAD^ template in the presence of Tof1-Csm3, stalled replication forks were almost entirely absent when Tof1-Csm3 was omitted (Figure 4A). Despite this dramatic reduction in fork stalling, collapse products accumulated at a similar rate, with and without Tof1-Csm3, likely because replisomes lacking Tof1-Csm3 progress at slower rates.^45^ This reduction in replication fork rate likely also explains why replication products are less intense in the absence of Tof1-Csm3. In contrast to Top1-cc^LEAD^, omission of Tof1-Csm3 did not trigger significant replication fork collapse at Top1-cc^LAG^ and stalled replication forks continued to accumulate across the reaction time course (Figure 4B). We conclude that the Tof1-Csm3 heterodimer plays a critical role in promoting replication fork stalling at Top1-cc^LEAD^, but is dispensable for fork stalling at Top1-cc^LAG^.

### Tof1-Csm3 modulates the architecture of stalled and collapsed replication forks

**Figure 5:**
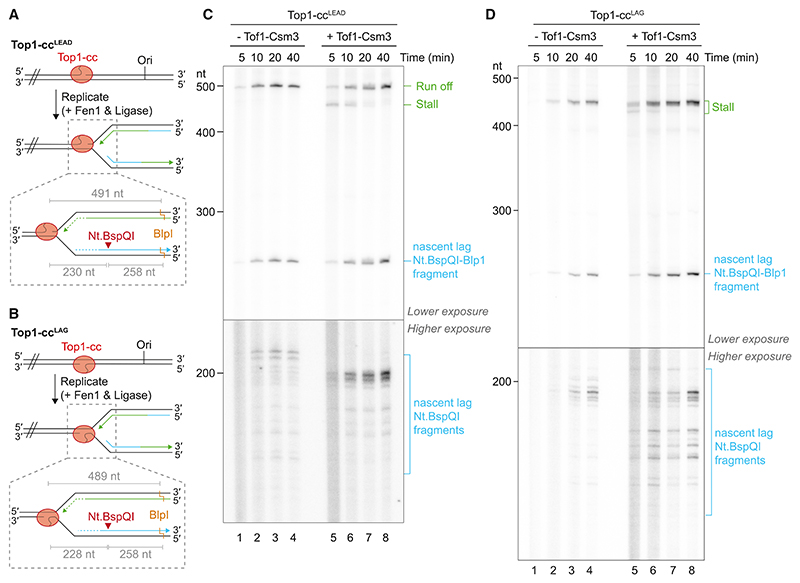
Tof1-Csm3 modulate the architecture of stalled and collapsed replication forks (A and B) Schematics of the post-reaction processing strategy used to liberate nascent leading and lagging strands from DNA replication reactions on Top1-cc^LEAD^ (A) and Top1-cc^LAG^ (B) templates for denaturing polyacrylamide gel analysis. Nascent leading strands are shown in green and matured lagging strands are shown in blue. Expected product sizes, indicated in gray, were calculated from the Top1-cc nick site to the respective site of strand cleavage for restriction enzymes. For clarity, the region of the template downstream of the dashed lines (//) is not shown. (C and D) Denaturing polyacrylamide gel analysis of replication products generated in time course reactions on Top1-cc^LEAD^ (C) and Top1-cc^LAG^ (D) templates, processed as indicated in (A) and (B). Two exposures of the same gel are shown to aid visualization of weaker bands (the full gels at each exposure are shown in Figure S6). Leading-strand products are labeled in green (runoff, stall) and lagging-strand products are shown in blue (Nt.BspQI-Blp1 fragment, Nt.BspQI fragments).

Because replication fork stalling at Top1-cc^LEAD^, but not Top1-cc^LAG^, was dependent on Tof1-Csm3 (Figure 4), we sought to examine more precisely how the complex modulates the disposition of stalled and collapsed replication forks. To do so, we developed a strategy to simultaneously map the 3′ ends of nascent leading strands and the 5′ ends of nascent lagging strands, the latter of which represent the final priming sites for lagging-strand replication at stalled and collapsed replication forks (Figures 5A and 5B). In the presence of Tof1-Csm3, leading strands were again observed to stall transiently at Top1-cc^LEAD^ before being extended to generate runoff products as the reaction progressed (Figure 5C, lanes 5–8 [stall/runoff], and Figures S5A and S5B). In contrast, no stall products were detected in the absence of Tof1-Csm3 and only runoff products were apparent, confirming that Tof1-Csm3 is essential for prolonged fork stalling at Top1-cc^LEAD^ (Figure 5C, lanes 1–4).

Analysis of lagging-strand products revealed that, in the presence of Tof1-Csm3, Okazaki fragments were not initiated within ~30 nt of the Top1-cc^LEAD^ nick (Figure 5C, lanes 5–8). However, when Tof1-Csm3 was omitted, the final initiation sites for lagging-strand synthesis were advanced to ~20 nt from the Top1-cc^LEAD^ nick and lagging-strand initiation sites were more evenly distributed across a broader region, consistent with replisomes failing to stall at the Top1-cc.^49^ Notably, the most prominent initiation sites observed in the presence of Tof1-Csm3, which are the closest to the Top1-cc, were utilized less frequently in the absence of Tof1-Csm3 (Figure 5C, compare lanes 4 and 8). This observation is consistent with a stochastic priming mechanism, whereby prolonged replication fork pausing increases the likelihood of lagging-strand initiation occurring close to the site of fork arrest. Finally, because most replication forks still collapse in the presence of Tof1-Csm3, the differing position of the final lagging-strand initiation sites, –/+ Tof1-Csm3, indicates that productive primer synthesis does not occur during Top1-cc displacement and subsequent leading-strand extension upon fork collapse.

At Top1-cc^LAG^, stalled leading strands accumulated across the reaction time course, with and without Tof1-Csm3 (Figures 5D, S5C, and S5D). However, the distribution of stall products differed between the two conditions. In the absence of Tof1-Csm3 stall products migrated as a single species that did not change as the reaction progressed. In the presence of Tof1-Csm3, leading strands appeared to stall in at least two positions before the shorter population was extended to the same position as when Tof1-Csm3 was omitted. This behavior is consistent with the broad distribution of stall products observed at early time points in Figure 2D. Moreover, it indicates that although replication forks do not collapse at Top1-cc^LAG^ in the absence of Tof1-Csm3, the behavior of the replisome as it collides with the topoisomerase is still modulated by Tof1-Csm3. The distribution of 5′ lagging-strand ends at Top1-cc^LAG^ further supports this conclusion. Figure 5D shows that in the absence of Tof1-Csm3, the final sites for lagging-strand initiation display a narrow distribution centered around ~30–35 nt from the Top1-cc nick, whereas a broader distribution is observed when Tof1-Csm3 is included in the reaction. This broader distribution is indicative of Tof1-Csm3-dependent heterogeneity in the initial position of replisome stalling at Top1-cc^LAG^. Collectively, these data illustrate that Tof1-Csm3 influences the disposition of collapsed replication forks at Top1-cc^LEAD^ and, although the complex is not required for fork stalling at Top1-cc^LAG^, it modulates the approach of the replisome to the Top1-cc and, consequently, the architecture of stalled replication forks.

### The Tof1 C terminus is dispensable for fork stalling at Top1-cc^LEAD^

A region of the flexible and largely unstructured ~450-amino-acid C terminus of Tof1 binds directly to Top1^50–53^ (Figure S6A). This interaction has been shown to limit replication fork rotation, likely via targeting Top1 to the replication fork to remove torsional strain.^52^ To evaluate whether this interaction contributes to replication fork stalling at Top1-ccs, we purified a Tof1-Csm3 complex where the flexible C terminus of Tof1 was removed (Tof1^1-783^-Csm3) (Figures S6A and S6B). Figures S6C–S6E show that Tof1^1-783^-Csm3 promoted fork stalling at Top1-cc^LEAD^ with a comparable efficiency to the wild-type complex, demonstrating that direct interaction between Top1 and the C terminus of Tof1 does not contribute to fork stalling in this context.

### Structures of stalled replication forks suggest replisome remodeling at Top1-cc^LAG^

**Figure 6:**
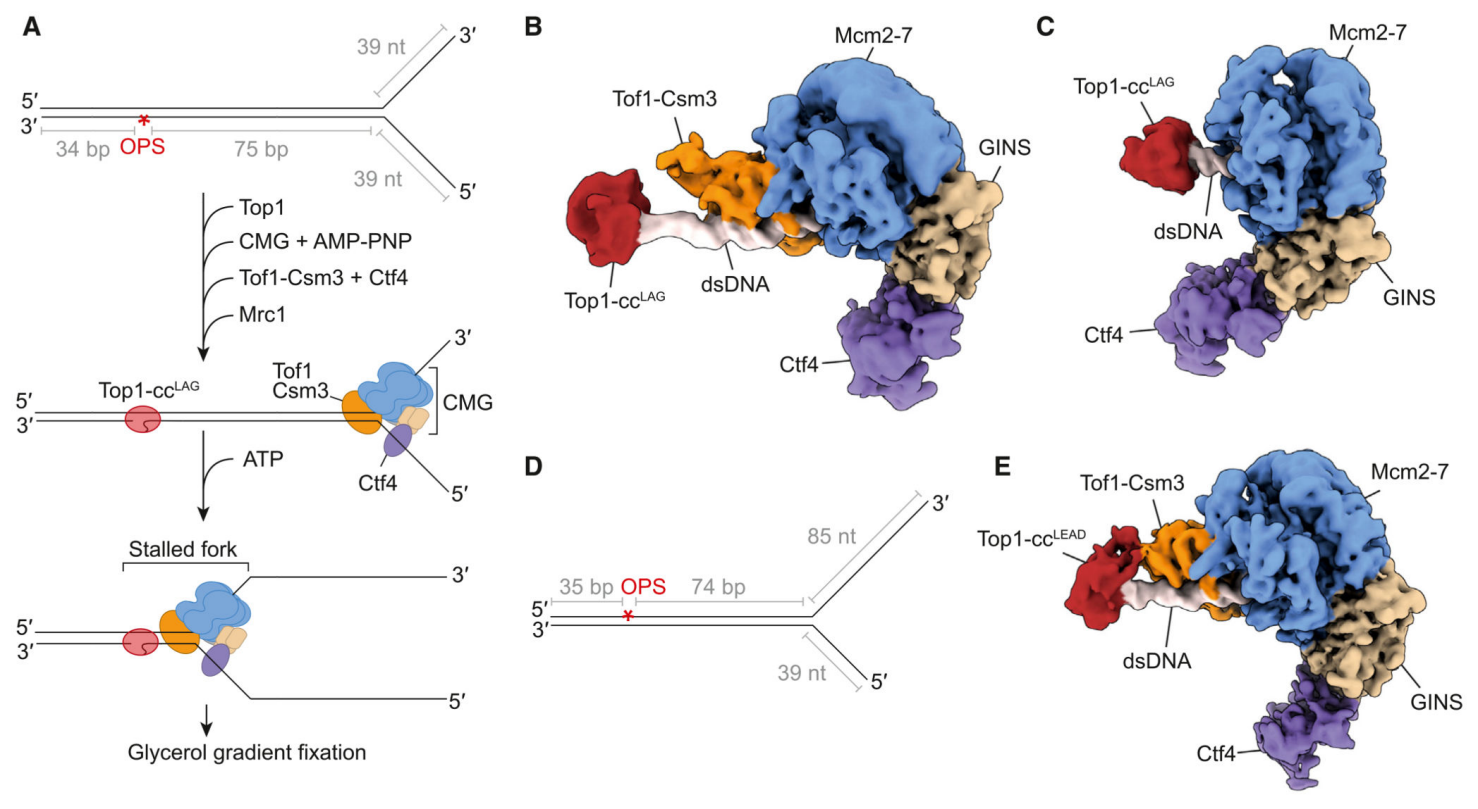
Structures of stalled replication forks suggest replisome remodeling at Top1-cc^LAG^ (A) Schematic of the model DNA replication fork and strategy used to obtain structures of replisomes stalled at Top1-cc^LAG^. The Top1-cc-forming OPS modification (indicated with a red asterisk) resides in duplex DNA 75 bp from the fork junction. (B) Cryo-EM map of a replisome stalled at Top1-cc^LAG^, with clear density for Tof1-Csm3 at the leading edge of the CMG helicase. (C) Cryo-EM map of a replisome stalled at Top1-cc^LAG^ lacking density for Tof1-Csm3. (D) Schematic of the model fork used for obtaining cryo-EM structures of replisomes stalled at Top1-cc^LEAD^. The Top1-cc-forming OPS modification (indicated with a red asterisk) resides in duplex DNA 74 bp from the fork junction. (E) Cryo-EM map of a replisome stalled at Top1-cc^LEAD^, with clear density for Tof1-Csm3 at the leading edge of the CMG helicase.

The final position of leading-strand arrest at Top1-cc^LAG^ remained unchanged, with and without Tof1-Csm3 (Figure 5D), which indicated that the footprint of stalled replisomes was the same whether these factors were included or not. This raised the intriguing possibility that Tof1-Csm3 was gradually lost from the replisome upon collision with Top1-cc^LAG^, which would enable CMG to advance closer toward the Top1-cc and allow the nascent leading strands to be extended. To explore this hypothesis further, we devised a strategy to image CMG complexes stalled at Top1-ccs by cryo-EM (Figure 6A). Replisome proteins and Top1 were incubated with a model replication fork that contained an OPS modification positioned 75 bp (for Top1-cc^LAG^) or 74 bp (for Top1-cc^LEAD^) from the fork junction within a 109-bp duplex region. The presence of the non-hydrolysable ATP analog, AMP-PNP, supports CMG loading onto leading-strand template ssDNA but not template unwinding. After complex formation, samples were incubated with ATP to facilitate unwinding and CMG translocation toward the Top1-cc to form stalled replication forks, which were isolated by glycerol gradient centrifugation and analyzed by cryo-EM.

The resulting density maps show replisomes consisting of CMG bound to substrate DNA, Ctf4, and Tof1-Csm3 at side-chain resolution (Figures S7–S9), resembling previous observations.^35^ Following extensive three-dimensional (3D) classification (Figure S7), we identified particle classes with additional density of appropriate shape and volume to accommodate Top1 positioned along the dsDNA ahead of the replisome. Because the OPS modification is sited 74–75 bp from the dsDNA-ssDNA junction of the substrate, Top1 density close to CMG is indicative of CMG translocation along the template and, therefore, these classes represent translocated replisome complexes that are stalled at Top1-ccs. Figures 6B and 6C show that at Top1-cc^LAG^, two distinct classes of stalled replication fork were observed. The first class displayed strong density for Tof1-Csm3 positioned on the N-terminal face of the MCM complex, consistent with prior cryo-EM structures^35^ (Figure 6B). Clear density for two helical turns of dsDNA (~20 bp) protrudes from the MCM complex, beyond Tof1-Csm3, to the DNA-bound Top1. In this configuration, CMG is presumably unable to advance up to the Top1-cc due to the presence of Tof1-Csm3 on the leading edge of the MCM ring. In the second class, density for Tof1-Csm3 is completely absent, with Top1 positioned directly ahead of the MCM N-terminal face (Figure 6C). Thus, these structures provide a likely explanation for why, in the presence of Tof1-Csm3, leading strands initially arrest further upstream of Top1-cc^LAG^. Although we cannot exclude the possibility that Tof1-Csm3 was never associated with a subset of the replisomes in our EM datasets, our structures, in conjunction with the observations from replication assays, suggest a model whereby Tof1-Csm3 is lost from the replisome after it initially stalls. This would enable CMG to advance further toward Top1-cc^LAG^, where it becomes stably stalled.

In contrast to Top1-cc^LAG^, CMG complexes stalled at Top1-cc^LEAD^ were all associated with Tof1-Csm3 (Figures 6D, 6E, and S8). This behavior is consistent with the essentiality of Tof1-Csm3 for fork stalling at Top1-cc^LEAD^ (Figures 4A and 5C). Collectively, these cryo-EM reconstructions further reinforce the concepts that the strand to which the topoisomerase is linked and the presence of Tof1-Csm3 at the head of the replisome are key factors in determining how the replisome initially responds to a Top1-cc.

## Discussion

**Figure 7:**
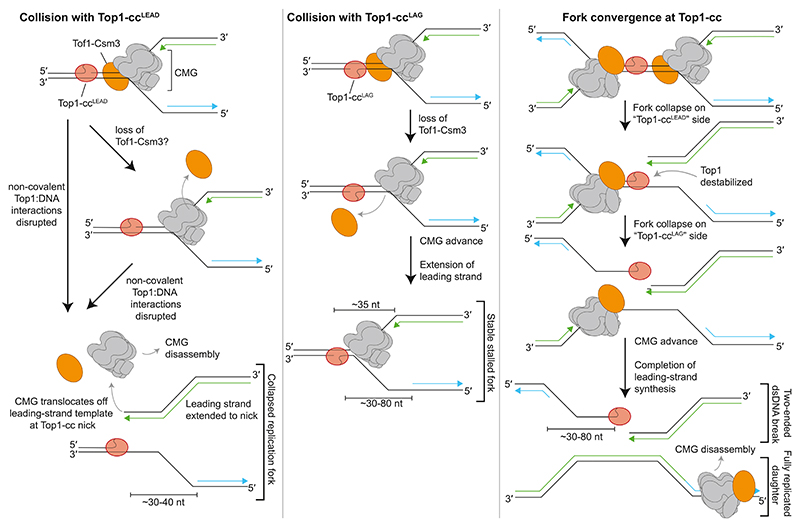
Model of how the replisome responds to Top1-ccs in different contexts and the influence of Tof1-Csm3 on such collisions For clarity, only the replisome factors CMG and Tof1-Csm3 are depicted.

Based on biochemical and structural analysis of replisome collisions with site-specific Top1-ccs, we propose the following model for replication fork stalling and collapse (Figure 7). When replisomes collide with Top1-cc^LEAD^, replication forks initially stall because the contacts between Top1 and DNA are maintained by the presence of Tof1-Csm3 on the leading edge of CMG. Eventually, Top1 DNA contacts are disrupted, potentially due to remodeling and/or displacement of Tof1-Csm3 from the replisome, causing CMG runoff and the collapse of the replication fork into a single-ended dsDNA break. At Top1-cc^LAG^, replication forks initially arrest with Tof1-Csm3 positioned between the Top1-cc and CMG. Similar to Top1-cc^LEAD^, Tof1-Csm3 is lost from the replisome and CMG advances to the Top1-cc. However, unlike at Top1-cc^LEAD^, forks do not collapse because CMG is unable to fully displace Top1 from dsDNA. When replication forks converge upon a Top1-cc, both forks initially stall. Collapse of the replication fork that encountered Top1 on the leading-strand template weakens the non-covalent interactions between Top1 and DNA so that Top1 can now be displaced by the replisome on the opposite side of the lesion. This double fork collapse generates a fully replicated daughter molecule and a two-ended dsDNA break, with Top1 attached to a 3′-terminated ssDNA tail on one side of the break.

The discovery that the core replisome initially pauses at a Top1-cc, regardless of the template strand that Top1 is attached to, is consistent with studies in yeast and human cells that did not detect dsDNA breaks at low doses of CPT—rather, replication forks were found to undergo fork reversal.^32^ This process slows replication and should allow more time for a replication-blocking Top1-cc to be resolved, either by ligation of the nick or via proteolysis of Top1 and subsequent repair of the remaining pep-tide-DNA crosslink. Because stalling of the replisome at the Top1-cc is a prerequisite for fork reversal, our data indicate that Tof1-Csm3 plays a crucial role in this process, specifically at Top1-cc^LEAD^. We propose that, mechanistically, it is the ability of Tof1-Csm3 to promote fork stalling at Top1-cc^LEAD^, thereby preventing rapid fork collapse, that underlies its role in the cellular tolerance of CPT.

Our cryo-EM structures and fork mapping experiments strongly suggest that Tof1-Csm3 promotes fork stalling by functioning as a buffer at the head of the replisome to prevent the replication fork junction at the leading edge of CMG from meeting Top1. The curved architecture of Tof1-Csm3 across the N-terminal domains of Mcms 2, 6, 4 and 7, and its interactions with parental dsDNA,^35^ are likely critical for this functionality. Consistent with this idea, cells harboring Tof1-Csm3 DNA-binding mutants were CPT sensitive.^35^ Due to the considerable heterogeneity in the positioning of Top1 relative to Tof1:Csm3 in our cryo-EM structures, it is unlikely that specific protein:protein interactions contribute significantly to fork stalling. Indeed, the Tof1 C terminus—which interacts directly with Top1—is dispensable for CPT resistance in yeast, or makes a minor contribution,^51,52^ and was not required for fork stalling at Top1-cc^LEAD^ in our experiments. Because Tof1 directly recruits Top1 to replication forks to remove topological stress,^52,54^ the replisome might frequently run into Top1 reaction intermediates, including transient Top1-ccs that form in the absence of exogenous Top1 poisons. We hypothesize that the fork stalling function of Tof1-Csm3 at Top1-cc^LEAD^ should prevent these collisions from forming highly toxic single-ended dsDNA breaks. Because the architecture of the core eukaryotic replisome, including the FPC, is conserved between yeast and human,^35,55^ TIMELESS-TIPIN likely confer resistance to CPT in human cells via the same mechanism as Tof1-Csm3. We hypothesize that the FPC has a conserved function at the front of the replisome, providing a physical buffer to maintain separation between the point of strand separation and a fork-stalling obstacle to stabilize replisomes.

During fork collapse at Top1-cc^LEAD^, we favor a model where Tof1-Csm3 rearrangement and/or displacement from the replisome precedes fork collapse because: forks rapidly collapse at Top1-cc^LEAD^ in the absence of Tof1-Csm3 and Tof1-Csm3 is lost from the replisome upon collision with Top1-cc^LAG^. Moreover, loss of Tof1-Csm3 would enable CMG to advance to Top1, where the action of strand separation would disrupt contacts between Top1 and leading-strand template DNA, triggering fork collapse. In support of this mechanism, experiments with nicked Top1 suicide substrates show that, 3′ of the nick site, 5-nt ssDNA fragments can be liberated after Top1 cleavage, indicating that this region of DNA—equivalent to the leading-strand template at Top1-cc^LEAD^—is not significantly stabilized by Top1.^56,57^ How Tof1-Csm3 is displaced from the replisome, whether there are additional factors that modulate Tof1-Csm3 stability, and whether Tof1-Csm3 displacement might be a general feature of prolonged replication fork stalling are subjects for future investigations. Interestingly, the TIMELESS-TIPIN complex is displaced from the replisome in response to redox changes in human cells.^58^ The absence of fork collapse at Top1-cc^LAG^ in unidirectional experiments, even after loss of Tof1-Csm3, is presumably because in this orientation, fork collapse will only occur if Top1 is fully dislodged from the template, which requires a greater number of non-covalent Top1:DNA contacts to be broken. It is possible that additional factors, such as accessory DNA helicases, might promote displacement of Top1 at stalled replication forks, which could increase the frequency and/or rate of replication fork collapse.

By reconstituting the earliest events that occur when replication forks collide with Top1-ccs, our work has revealed a diverse range of outcomes that are heavily influenced by core replisome components and the orientation of the Top1-cc. Gaining deeper insight into the factors and mechanisms that modulate fork stalling and collapse at Top1-ccs should help to better tailor the use of Top1 inhibitors in the clinic.

### Limitations of the study

Although our study gives valuable insights into the response of the core eukaryotic replisome to Top1-ccs, additional factors not included in our experiments are likely to have important roles in response to these lesions. These may include accessory helicases, which have been shown to assist replisomes in overcoming proteinaceous barriers, and components of the DNA replication checkpoint that stabilize stalled replication forks and promote DNA repair. Second, in our experiments, we have used reconstituted budding yeast replisomes. Although these replisomes are highly similar, both structurally and functionally, to those assembled with human proteins, further work is necessary to establish precisely how the human replisome responds when it collides with a Top1-cc.

## Star ★ Methods

Detailed methods are provided in the online version of this paper and include the following:

- KEY RESOURCES TABLE
- RESOURCE AVAILABILITY
  ◦ Lead contact
  Materials availability
  Data and code availability
- EXPERIMENTAL MODEL AND STUDY PARTICIPANT DETAILS
- METHOD DETAILS
  - Templates for in vitro DNA replication reactions
  - Generation of DNA constructs containing 5^′^ -bridging phosphoro-thioate modifications
  - In vitro DNA replication reactions
  - Post-reaction sample processing
  - Protein purification
  - Preparation of fork DNA for cryo-EM
  - Modified lagging strand (Top1-cc^LAG^ fork):
  - Modified leading strand (Top1-cc^LEAD^ fork):
  - Reconstitution of replisome encounters with lagging- or leading-strand Top1-cc lesions for cryo-EM
  - Cryo-EM grid preparation
  - Cryo-EM data collection
  - Cryo-EM data processing
- QUANTIFICATION AND STATISTICAL ANALYSIS

## Star ★ Methods

### Key Resources Table

**Table:** 

REAGENT or RESOURCE	SOURCE	IDENTIFIER
Bacterial strains		
*E. coli* 5-alpha Competent (High Efficiency)	New England Biolabs	Cat# C2987H
*E. coli* Rosetta™ 2(DE3) strain: F-ompT hsdSB(rB- mB-) gal dcm (DE3) pRARE2 (CamR)	Novagen / Merck Millipore	Cat# 71400
Chemicals, peptides, and recombinant proteins		
dNTP set	Invitrogen	Cat# 10297018
NTP set	Invitrogen	Cat# R0481
[alpha-P32]dATP	Hartmann analytic	Cat# SCP-203
[alpha-P32]dCTP	Hartmann analytic	Cat# SCP-205
[gamma-P32]ATP	Hartmann analytic	Cat# SRP-301
NP-40-S	Roche	Cat# 11754599001
TWEEN® 20	Sigma Aldrich	Cat# P8341
cOmplete™, Mini, EDTA-free protease inhibitor cocktail	Sigma Aldrich	Cat# 11873580001
Bovine Serum Albumin (BSA)	Invitrogen	Cat# AM2616
Phusion® High-Fidelity DNA Polymerase	New England Biolabs	Cat# E0553
Proteinase K	New England Biolabs	Cat# P8107
Nt.BbvCI	New England Biolabs	Cat# R0632
BamHI	New England Biolabs	Cat# R0136
PstI	New England Biolabs	Cat# R0140
Nt.BspQI	New England Biolabs	Cat# R0644
NheI-HF®	New England Biolabs	Cat# R3131
SmaI	New England Biolabs	Cat# R0141
BlpI	New England Biolabs	Cat# R0585
T4 PNK	New England Biolabs	Cat# M0201
Sepharose™ 4B	Sigma Aldrich	Cat# 4B200
T4 DNA Ligase	New England Biolabs	M0202
Phenol:chloroform:isoamyl (alcohol 25:24:1 saturated with TE)	Sigma Aldrich	P2069
SeaKem® LE Agarose	Lonza	Cat# 50004
1% Ethidium bromide solution	Sigma Aldrich	Cat# 46067
Adenosine 5’-(β,γ-imido)triphosphate lithium salt hydrate (AMP-PNP)	Sigma Aldrich	Cat# A2647
Glutaraldehyde	Sigma Aldrich	Cat# G5882
Adenosine 5’-triphosphate (ATP) for protein purification	Sigma Aldrich	Cat# A7699
Cesium Chloride (CsCl)	Sigma Aldrich	Cat# C4036
Anti-FLAG M2 affinity gel	Sigma	Cat# A2220
Bio-Gel HT (Hydrated) Hydroxyapatite	Bio-Rad	Cat# 130-0150
Calmodulin-Sepharose 4B	GE Healthcare	Cat# 17-0529-01
StrepTactin Superflow high-capacity resin	IBA life sciences	Cat# 2-1208-002
Recombinant Proteins—see also Table S3		N/A
Cdt1-Mcm2-7	Coster et al.^59^	N/A
Cdc6 (Expressed in E. coli)	Coster et al.^59^	N/A
DDK	On et al.^60^	N/A
ORC	Frigola et al.^61^	N/A
S-CDK (*clbΔ1-100*^62^)	Hill et al.^62^	N/A
Dpb11	Yeeles et al.^44^	N/A
GINS	Yeeles et al.^44^	N/A
Cdc45	Yeeles et al.^44^	N/A
Mcm10	Yeeles et al.^44^	N/A
Polymerase ε	Yeeles et al.^44^	N/A
Ctf4	Yeeles et al.^44^	N/A
RPA	Baretić et al.^35^	N/A
RFC	Yeeles et al.^45^	N/A
PCNA	Yeeles et al.^45^	N/A
Tof1-Csm3	Baretić et al.^35^	N/A
Tof1^1-783^-Csm3	This study	N/A
Polymerase α-primase	Yeeles et al.^45^	N/A
Polymerase δ	Yeeles et al.^45^	N/A
Mrc1	Baretić et al.^35^	N/A
Sld3/7	Yeeles et al.^44^	N/A
Sld2	Yeeles et al.^44^	N/A
Fen1	Guilliam and Yeeles.^63^	N/A
Cdc9 (Ligase)	Guilliam and Yeeles.^63^	N/A
Top1	Yeeles et al.^45^ (With different storage buffer).	N/A
CMG	Baretić et al.^35^	N/A
Deposited data		
Replisome stalled at a lagging-strand Topoisomerase 1 cleavage complex with Tof1-Csm3	This study	EMD-50392
Replisome stalled at a lagging-strand Topoisomerase 1 cleavage complex missing Tof1-Csm3	This study	EMD-50393
Replisome stalled at a leading-strand Topoisomerase 1 cleavage complex	This study	EMD-50395
Unprocessed and uncropped gel scans (deposited to Mendeley Data)	This study	https://doi.org/10.17632/dy9mt64mv7.1
Experimental models: Organisms/strains		
*S. cerevisiae strains*		N/A
*yAM33 (Cdt1-Mcm2-7 purification)*	Coster et al.^59^	N/A
*ySDORC (ORC purification)*	Frigola et al.^61^	N/A
*ySDK8 (DDK purification)*	On et al.^60^	N/A
*yTD6 (Sld3/7 purification)*	Yeeles et al.^44^	N/A
*yTD8 (Sld2 purification)*	Yeeles et al.^44^	N/A
*yJY13 (Cdc45 purification)*	Yeeles et al.^44^	N/A
*yJY26 (Dpb11 purification)*	Yeeles et al.^44^	N/A
*yAJ2 (Pol epsilon purification)*	Yeeles et al.^44^	N/A
*yAE88 (S-CDK purification)*	Hill et al.^62^	N/A
*yAE95 (Pol alpha purification)*	Hill et al.^62^	N/A
*yAE40 (Ctf4 purification)*	Yeeles et al.^44^	N/A
*yJY106 (RPA purification)*	Baretić et al.^35^	N/A
*yJY32 (Mrc1 purification)*	Yeeles et al.^45^	N/A
*yAE48 (Tof1-Csm3 purification)*	Yeeles et al.^45^	N/A
*yAE41 (RFC purification)*	Yeeles et al.^45^	N/A
*yJY31 (Fen1 purification)*	Guilliam et al.^63^	N/A
*yJY33 (Ligase purification)*	Guilliam et al.^63^	N/A
*yAE42 (Top1 purification)*	Yeeles et al.^45^	N/A
*yAE34 (Pol delta purification)*	Yeeles et al.^45^	N/A
*yJY197 (CMG purification)*	Jenkyn-Bedford et al.^64^	N/A
yRW9 (Tof1^1-783^-Csm3 purification) Genotype: *MATa ade2-1 ura3-1 his3-11,15 trp1-1 leu2-3, 112 can1-100 bar1::Hyg pep4::KanMX ura3::URA Gal1-10 CBP-Csm3/tof1 1-783*	This study	N/A
Oligonucleotides		
See Table S2 for details of oligonucleotides	This study	N/A
Other		
Amicon Ultra Centrifugal Filter Units	Millipore	Cat# UFC901096
QUANTIFOIL Copper 400 mesh R2/2 holey carbon TEM grids	Electron Microscopy Sciences	Cat# Q450CR2
HiTrap Blue HP	GE Healthcare	Cat# 17-0412-01
HiTrap DEAE Fast Flow	GE Healthcare	Cat# 17-5055-01
HiTrap Heparin HP	GE Healthcare	Cat# 17-0406-01
HiTrap SP HP	GE Healthcare	Cat# 29-0513-24
HiTrap SP FF	GE Healthcare	Cat# 29-0513-24
IgG Sepharose Fast Flow	GE Healthcare	Cat# 17-0969-01
MonoQ PC 1.6/5	GE Healthcare	Cat# 17-0671-01
MonoS 5/50 GL	GE Healthcare	Cat# 17-5168-01
Ni-NTA Agarose	QIAGEN	Cat# 30210
Superdex 200 Increase 10/300 GL	GE Healthcare	Cat# 28-9909-44
Superose™ 6 Increase 10/300 GL	GE Healthcare	Cat# 29-0915-96
illustra MicroSpinG-50 columns	Cytiva	Cat# GE27-5330
Criterion XT 4-12% Bis-Tris precast gels	BioRad	Cat# 3450124
NuPAGE™ 4-12% Bis-Tris precast gels	Thermo Fisher	Cat# NPO323box
Whatman 3 MM paper	Cytivia	Cat# 11895375
BAS-IP MS phosphor screen	Cytivia	Cat# 28956474
Amersham Hyperfilm MP	Cytivia	Cat# 28906842
Recombinant DNA		
See also Table S1		N/A
ZN5_BspQI_Rem	This study	N/A
vRW8	This study	N/A
vRW11	This study	N/A
vRW12	This study	N/A
vRW23	This study	N/A
vRW24	This study	N/A
vRW19	This study	N/A
Software and algorithms		
Photoshop 2020	Adobe	https://www.adobe.com/uk/products/photoshop.html
Amersham Typhoon (1.1.0.7)	Cytiva	N/A
EPU (v2)	ThermoFisher Scientific (FEI)	https://www.fei.com/software/epu-automated-single-particles-software-for-life-sciences
RELION (v4)	Sjors Scheres (Medical Research Council Laboratory of Molecular Biology)	https://www3.mrc-lmb.cam.ac.uk/relion/
cryoSPARC (v3 & v4)	Structura Biotechnology	https://cryosparc.com/updates
CTFFIND-4.1	The Grigorieff Lab	https://grigoriefflab.umassmed.edu/ct1find4
Pymol (v2.5)	Schrodinger	https://pymol.org
Epson Scan 3.9.3.0EN	Seiko Epson Corporation	https://www.epson.co.uk

### Resource Availability

#### Lead contact

Further information and requests for resources and reagents should be directed to and will be fulfilled by the lead contact, Joseph Yeeles (jyeeles@mrc-lmb.cam.ac.uk).

#### Materials availability

Budding yeast strains and protein expression plasmids will be made available on request.

#### Data and code availability

- Cryo-EM density maps have been deposited in the Electron Microscopy Data Bank (EMDB) and are publicly available as of the date of publication. Accession numbers are listed in the key resources table. Unprocessed images of the gels featured in this manuscript have been deposited at Mendeley and are publicly available as of the date of publication. The DOI is listed in the key resources table.
- This paper does not report original code.
- Any additional information required to reanalyze the data reported in this paper is available from the lead contact upon request.

#### Experimental Model And Study Participant Details

*S. cerevisiae* strains used for protein purification can be found in the Key Resources Table.

### Method Details

#### Templates for in vitro DNA replication reactions

The DNA replication template designed to trap a Top1-cc (vRW8) was generated by introduction of a Top1-binding cassette into ZN5_BspQI_Rem, a modified form of ZN5^36^ with the BspQI site removed. Specifically, two oligos (ZN3_Top1_3_Top & ZN3_Top1_3_Bot) were annealed and 5^′^ phosphorylated with T4 PNK (NEB #M0201S), and the duplex ligated into the BamHI and PstI sites of ZN5_BspQI_Rem. This cassette contained a 29-bp sequence (5^′^-TCAGCAAAAAGACTTCGAAAAATTTTTCC-3^′^) with similarity to a previously described Top1 binding sequence^37^ flanked by two BbvCI sites. The 29-bp Top1 binding sequence was subsequently replaced with an oligo of the same sequence but containing a 5^′^ bridging phosphorothioate (oRW30) to prevent reversal of the Top1 covalent complexes^38^ (see “generation of DNA constructs containing 5’-bridging phos-phorothioate modifications”).

Additional templates incorporating BspQI sites for mapping of nascent leading- or lagging-strand replication products were gener-ated as follows: to generate vector vRW11 (used for mapping nascent leading strands at a leading-strand Top1-cc: Figures 2B and S3B) vRW8 was modified by site-directed mutagenesis using oligos oRW40 & oRW41. To generate vector vRW12 (used for mapping nascent lagging strands at a leading-strand Top1-cc: Figure 5C) vRW8 was modified by site-directed mutagenesis using oRW42 & oRW43.

To generate templates in which the Top1-binding sequence resided in the bottom strand (lagging-strand Top1-cc templates), the Top1-binding cassette in vRW11 and vRW12 was replaced with a cassette in which the Top1-binding sequence (including the BbvCI sites for introduction of an OPS modification) was inverted. This generated vRW23 (used for all experiments on Top1-cc^LAG^ templates except Figure 5D) and vRW24 (used for mapping nascent lagging strands at Top1-cc^LAG^: Figure 5D) respectively.

#### Generation of DNA constructs containing 5′-bridging phosphorothioate modifications

To introduce the site-specific 5^′^ -bridging phosphorothioate modification into DNA replication templates, we used the same approach as previously described for introducing site-specific damage into DNA.^36^ First, DNA was purified from *E. coli* using QIAGEN Maxiprep kits (#12163) according to the manufacturer’s instructions. Four digestions were then set up, each containing 160 μg of maxiprep DNA, 1X CutSmart buffer (NEB #B6004) and 21 μl Nt.BbvCI (NEB #R0632) in a total volume of 400 μl for 2 hours at 37°C. A further 5 μl of Nt.BbvCI was added to each digestion and continued for 1 hour before the reactions were stopped by the addition of EDTA (pH 8.0) to 50 mM. Competitor oligo (oRW25 or oRW97), complementary to the to the nicked region, was added to each tube in 1000-fold excess over the plasmid DNA. The DNA was heated to 63°C for 20 minutes, before digestion of proteins by addition of SDS/ Proteinase K. Gapped DNA was purified from the competitor oligo by loading onto a Sepharose 4B (Sigma-Aldrich #4B200) column equilibrated in 5 mM Tris-HCl (pH 8.0), 0.1 mM EDTA in a siliconized 1 m x 1 cm econo-column (Biorad #7371091). The column was run under gravity flow and peak fractions containing the gapped plasmid were concentrated 10-fold in a vacuum concentrator (ScanVac ScanSpeed 40 Centrifuge). Gapped DNA (100-150 μg) was annealed to a 20-fold molar excess of oRW30. DNA was then ligated in 1X T4 DNA Ligase buffer (NEB #B0202), 0.5 mM ATP and 2 mM Mg(OAc)_2_, with 110 units of T4 DNA ligase (NEB #M0202M) per μg of DNA, at 16°C overnight. Proteins were digested by addition of SDS/proteinase K and the DNA extracted with phenol:chloroform:isoamyl alcohol 25:24:1 saturated with TE (Sigma-Aldrich P2069). The aqueous phase was dialysed against 2 L Tris-HCl pH 8.0, 5 mM EDTA (TE) for 1 h and the ligated material was purified by cesium chloride gradient purification.

#### In vitro DNA replication reactions

DNA replication reactions were performed essentially as previously described.^44,45^ A loading reaction was first set up in which 3 nM template DNA (linearised with AhdI (NEB) or circular) was incubated with 75 nM Cdt1-Mcm2-7, 45 nM Cdc6, 50 nM DDK and 20 nM ORC, in 1 X Replication Buffer (25 mM HEPES-KOH pH 7.6, 100 mM potassium glutamate, 10 mM Mg(OAc)_2_, 1 mM DTT, 0.02% NP-40-S, 0.1 mg/mL BSA), 5 mM ATP and 40 mM KCl. MCM loading was carried out at 24°C for 10 min before addition of S-CDK to 120 nM. The loading reaction was further incubated for 5 min at 24°C before being diluted 4-fold into a pre-equilibrated replication mix. Topoisomerase 1 was added to the replication mix to 10 nM to generate Top1-ccs, and the reactions were equilibrated at 30°C (typically <1 min). A cocktail of proteins was then added to initiate replication and were used at the following final concentrations: 30 nM Dpb11, 100 nM GINS, 30 nM Cdc45, 10 nM Mcm10, 20 nM DNA polymerase ε, 20 nM Ctf4, 100 nM RPA, 20 nM RFC, 20 nM PCNA, 20 nM Tof1-Csm3, 20 nM DNA polymerase α-primase, 5 nM DNA polymerase δ, 10 nM Mrc1, 12.5 nM Sld3/7, 20 nM Sld2. For reactions containing Fen1 & Cdc9 (Ligase), these were used at 20 nM final each. The final composition of replication reactions was as follows: 1 X Replication Buffer, 200 μM each CTP/GTP/TTP, 30 μM each dCTP/dCTP/dTTP/dATP, 3 mM ATP, 33 nM α-[32P]-dCTP or α-[32P]-dATP, 250 mM potassium glutamate (100 mM contributed by 1 x Replication Buffer and 150 mM added to the replication mix), and proteins. For experiments without Tof1-Csm3, the potassium glutamate concentration was reduced to 150 mM final and the concentration of Mrc1 was raised to 20 nM.

#### Post-reaction sample processing

For denaturing agarose gel analysis, time points (10 μl) were taken from replication reactions and quenched by addition of EDTA (pH 8.0) to 25 mM, before removal of unincorporated nucleotides with illustra MicroSpinG-50 columns (GE Healthcare). EDTA was added to 25 mM and 1/10 volume alkaline gel loading dye added (0.5 M NaOH, 10% sucrose and ~0.04% xylene cyanol). Samples were run on 0.6% agarose gels prepared and run in 30 mM NaOH, 2 mM EDTA at 24V overnight.

For native agarose gel analysis, samples were minimally processed before loading onto gels. Time points (5-10 μl) were taken from replication reactions and quenched by addition of EDTA to 25 mM, before addition of 1/5 volume of native loading dye (20 mM Tris-HCl (pH 8.0), 50 mM EDTA, 10% Ficoll 400, 2% N-Lauroylsarcosine sodium salt solution). Samples were loaded onto vertical 1% agarose gels made and run in 50 mM Tris-HCl (pH 7.9), 40 mM sodium acetate, 1 mM EDTA (pH 8.0), at 24V overnight.

For two-dimensional (2D) gel analysis, time points (20 μl) were taken from replication reactions and quenched by addition of EDTA to 25 mM. The sample was split, and 5% of the sample was loaded in one lane of a 1% native agarose gel for analysis. The remaining sample was then loaded in another lane on the same gel. The latter lane was excised from the native gel, embedded into a 0.8% denaturing agarose gel and run at 27V overnight.

For analysis on denaturing polyacrylamide gels, 20 μl time points were taken from replication reactions and quenched by addition of EDTA to 25 mM, before digestion of proteins with SDS/proteinase K and phenol chloroform extraction. The aqueous phase was passed over illustra MicroSpin G-50 columns to remove unincorporated nucleotides. At this stage any required restriction digests were performed as indicated in the figures and figure legends, according to manufacturer’s instructions (all restriction enzymes were supplied by NEB). Where two or more enzymes with different reaction buffers were used, digests were stopped by addition of EDTA to 50 mM before buffer exchange with illustra MicroSpinG-50 columns between digests. After all required restriction enzyme digests were complete, samples were proteinase K-treated as above before phenol chloroform extraction and ethanol precipitation of the DNA. Each reaction was resuspended in 4 μl TE and prior to gel loading, an equal volume of loading buffer (80% formamide, 0.05% SDS, 10 mM EDTA, 100 mM NaCl, 0.04% xylene cyanol, 0.04% bromophenol blue) was added. Samples were boiled for 5 min and products were resolved on a 40 cm x 20 cm 6% polyacrylamide (Bis-Acrylamide 19:1 – Fisher Scientific), 7 M urea denaturing gel, run in 1x Tris-Borate-EDTA buffer (TBE) for 150 min at 40 W.

Native agarose and denaturing polyacrylamide gels were dried onto 3MM chromatography paper (GE Healthcare) before imaging. Alkaline agarose gels were fixed with 2 x washes in 5% trichloroacetic acid and subsequently neutralised in 1M Tris-HCl (pH 8.0) prior to drying. Dried gels were exposed on BAS-IP MS Storage Phosphor Screens (GE Healthcare) and visualised on a Typhoon phosphorimager (GE Healthcare).

#### Protein purification

For protein purification strategies see Table S3.

#### Preparation of fork DNA for cryo-EM

For the reconstitution of replisomes at lagging- or leading-strand lesions, the 29 nt 5′-bridging phosphorothioate-containing oligo (oRW30) was incorporated into a longer (148 and 193 nt respectively) DNA oligomer by splint oligo-guided T4 ligation. Ligation reactions were treated with Proteinase K and SDS, separated by denaturing PAGE (7.5 M urea, 6 % polyacrylamide), and desired bands were identified by UV-shadowing and excised. Gel pieces were shredded, eluted into TE, and the DNA was ethanol-precipitated and re-dissolved in TE.

Complementary leading- and lagging-strand oligomers were obtained commercially from Integrated DNA Technologies and annealed by heating and gradual cooling from 80°C to room temperature in 25 mM HEPES-NaOH, pH 7.5, 150 mM NaOAc, 0.5 mM TCEP, 2 mM Mg(OAc)_2_) to obtain forked DNA templates for replisome reconstitution.

See Table S2 for sequences of all oligos used in construction of fork DNA.

The final oligomer sequences used for fork preparation were (5^′^ -3^′^):

#### Modified lagging strand (Top1-cc^LAG^ fork)

GGCAGGCAGGCAGGCAGGCAGGCAGGCAGGCAGGCAGGCACACACTCTCCAATTCTCTAATCACTTACCATCACTTCCTACTCT ATGGTTTATTGACAATCAGCAAAAAGACTT^CGAAAAATTTTTCCTTACTTATACACTGTTACAT (with ^ demarking the position of the 5^′^ -bridging phosphorothioate in the oligo backbone).

Leading strand (Top1-cc^LAG^ fork):

ATGTAACAGTGTATAAGTAAGGAAAAATTTTTCGAAGTCTTTTTGCTGATTGTCAATAAACCATAGAGTAGGAAGTGATGGTAAGTG ATTAGAGAATTGGAGAGTGTG(T)_40_-

#### Modified leading strand (Top1-cc^LEAD^ fork)

ATGTAACAGTGTATAAGTAATCAGCAAAAAGACTT^CGAAAAATTTTTCCTTGTCAATAAACCATAGAGTAGGAAGTGATGGTAAGTG ATTAGAGAATTGGAGAGTGTGTTTTTTTTTTTTTTTTTTTTTTTTTTTTTTTTTTTCGATAGGCCGATAGATTTTTTTTTTTTTTTTTTTTTTT TTTTTTTTTTTT (with ^ demarking the position of the 5′-bridging phosphorothioate in the oligo backbone).

Lagging strand (Top1-cc^LEAD^ fork):

GGCAGGCAGGCAGGCAGGCAGGCAGGCAGGCAGGCAGGCCACACTCTCCAATTCTCTAATCACTTACCATCACTTCCTACTCT

ATGGTTTATTGACAAGGAAAAATTTTTCGAAGTCTTTTTGCTGATTACTTATACACTGTTACAT

#### Reconstitution of replisome encounters with lagging- or leading-strand Top1-cc lesions for cryo-EM

The pre-annealed forked DNA templates were first incubated with Top1 in reconstitution buffer (25 mM HEPES-NaOH (pH 7.5), 150 mM NaOAc, 10 mM Mg(OAc)_2_, 0.5 mM TCEP) for 15 min at room temperature and 30 min on ice. Replisomes were then reconstituted as described previously^35^ (Figures S7 and S8). In brief, CMG and 0.5 mM AMP-PNP were incubated with the fork template on ice for 1h. Ctf4 and Tof1-Csm3 were then added, followed by 30 min incubation on ice, before Mrc1 was added and incubated another 30 min. The reconstitution reaction was adjusted with reconstitution buffer to 180 μL and mixed with 20 μL 50 mM ATP in reconstitution buffer + 20 mM MgOAc. After 1h of incubation at 15°C (for the lagging-strand Top1-cc template) or 20 min at room temperature (for leading-strand Top1-cc template), 180 μL of the reactions were subjected to cross-linking glycerol gradient sedimentation as described previously,^35^ while the remaining 20 μL were diluted to 180 μL and loaded onto a 10-30% glycerol gradient without crosslinker. Gradients were fractionated and SDS-PAGE gel analysis (Figures S7 and S8) was used to identify 4 peak fractions in the gradient containing crosslinker. These fractions were pooled, buffer-exchanged into 25 mM HEPES-NaOH (pH 7.5), 150 mM NaOAc, 0.5 mM TCEP, 0.005% Tween-20, 0.1 mM AMP-PNP and concentrated to 40 μL for cryo-EM grid preparation.

#### Cryo-EM grid preparation

Quantifoil R2/2, Cu-400 mesh cryo-EM grids pre-coated with a 2 nm film of amorphous carbon (electron microscopy sciences) were glow discharged for 5 s in a plasma current of 15 mA at 0.39 mBar (PELCO easiGlow). 3 μl sample was applied to the darker carbon side and incubated for 30 s at 4°C before manually blotting with filter paper for 10 s and plunge-freezing in liquid ethane.

### Cryo-EM data collection

#### Replisome on a Top1 lagging-strand lesion

A dataset of 7,068 multi-frame movies was collected on a 300 kV FEI Titan Krios equipped with a Gatan K3 detector operated in electron counting mode with the BioQuantum energy filter at a slit width of 20 eV. Data were collected at 81,000-fold magnification and in super-resolution mode (bin 2) at an effective pixel size of 0.93 Å/pixel over a defocus range of -0.8 to -3.0 μm. Movies were dosefractionated into 40 fractions over a 1 s exposure with a total dose of 34.8 e^-^/Å 2.

#### Replisome on a Top1 leading-strand lesion

A dataset of 15,284 multi-frame movies was collected as above, with a total dose of 39.9 e^-^/Å 2 over a 1.3 s exposure.

### Cryo-EM data processing

#### Replisome on a Top1 lagging-strand lesion

A schematic of the processing pipeline described here is outlined in Figure S7C. The 7,068 movies were patch motion corrected in CryoSPARC v4^65^ and 6,172 micrographs were selected via manual curation after Patch CTF Estimation. Figure S7D shows a representative micrograph after Patch CTF estimation, low-pass filtered at 3 Å. Particles were picked using Blob Picker on 500 micrographs, extracted, and 2D-classified to generate class averages for template-based picking on all micrographs. 4.06 M particles were extracted (372 Å box size, binned 2-fold) subjected to 2D classification. 6 classes with clear replisome-like features and containing 394,180 particles were selected (Figure S7E), used for Ab-Initio Reconstruction and one round of Heterogeneous Refinement. Particles of the best class were extracted at their original pixel size, refined via Non-uniform Refinement, and subjected to Local Motion Correction and CTF refinement. 3D classification with a soft mask around the density for the fork protection complex was performed to separate replisomes containing Tof1-Csm3 from replisomes lacking Tof1-Csm3. The reconstruction from 149,925 particles containing clear signal for Tof1-Csm3 (Figure S7F) were further classified during two iterative rounds of 3D Classification with a soft mask around the weak signal in the area ahead of Tof1-Csm3, isolating a final set of 5,886 particles that were refined via Homo-geneous Refinement to 6.2 Å resolution and locally filtered using the estimated local resolution.

Because the 3D class lacking Tof1-Csm3 had a comparatively small particle number, all non-junk particles from 2D classification were subjected to two iterative rounds of Heterogeneous Refinement, followed by 3D Classification with a soft mask around the density for the fork protection complex as described above. The reconstruction from the resulting particle set lacking Tof1-Csm3 (92,203 particles) was still engaged at fork DNA which extended beyond the MCM zinc finger domains. Two iterative rounds of 3D Classification with a large mask around the weak signal were performed to enrich the signal of the Top1-cc. The final particle set of 10,017 particles was refined homogeneously to 4.7 Å resolution and the map was locally filtered as described above.

#### Replisome on a Top1 leading-strand lesion

A schematic of the processing pipeline described here is outlined in Figure S8C. 15,284 multi-frame movies were motion corrected in RELION-4^66^ using RELION’s own implementation of a MotionCor2-like program. Aligned and dose-weighted micrographs were then migrated to CryoSPARC v4.^65^ Figure S8D shows a representative micrograph after Patch CTF estimation, low-pass filtered at 3 Å.

Particles were picked with Blob Picker in CryoSPARC, providing a particle diameter range of 150-400 Å, and roughly 4.46 M particles were extracted with a box size of 450 px (418.4 Å), binned 3-fold to 150 px (2.79 Å /px) during extraction, and subjected to 2D classification. Particles with class averages displaying replisome-like features were selected (Figure S8E) and a subset of 200,000 particles was used to generate initial 3D models by Ab-Initio Reconstruction. A 50,000-particle subset from the excluded 2D classes was used to generate a second set of 3D-references. All extracted particles were then classified in 3D via Heterogeneous Refinement providing all generated 3D volumes. Roughly 1.86 M replisome particles were selected and subjected to a second round of Hetero-geneous Refinement, providing the refined replisome map three times. The 3D-class with density for Tof1-Csm3 and duplex DNA was selected (1.07 M particles) and re-extracted with a box size of 536 Å, downsampled 2-fold to a pixel size of 1.86 Å. The particles were refined via Homogeneous Refinement and classified with a soft mask around the MCM C-tier (generated in UCSF Chimera^67^ and CryoSPARC Volume Tools) via 3D Classification without alignment (using PCA initialisation mode, 8 classes, 6 Å target resolution). A subset of roughly 70,000 particles displayed clear single-stranded DNA in the MCM pore with the AAA+ domains of the MCM C-tier engaged (Figure S8F). These particles were further classified with a soft mask around the density for the parental DNA duplex that reaches beyond the Tof1-Csm3. Two separate 3D Classification jobs were run using either weighted back projection or hard classification. Particles with the strongest signal for Top1 on the parental DNA duplex were combined and classified again as above. The final set of 7,702 particles was homogeneously refined to 4.9 Å resolution and locally filtered using the estimated local resolution.

### QUantification And Statistical Analysis

No quantification or statistical analysis were performed in this manuscript.

## Supplementary Material

**Supplemental Information**

Supplemental information can be found online at https://doi.org/10.1016/j.molcel.2024.08.004.

Document S1. Figures S1–S9, Tables S1–S3, and supplemental references.

Document S2. Article plus supplemental information.

## Highlights

- Reconstitution of collisions between *in vitro*-assembled replisomes and Top1-ccs
- The template strand that a Top1-cc is crosslinked to heavily influences collisions
- Fork protection factors Tof1-Csm3 mediate fork pausing at a leading-strand Top1-cc
- Cryo-EM and biochemical data suggest replisome remodeling upon Top1-cc collision

## References

[1] Zeman MK, Cimprich KA (2014). Causes and consequences of replication stress. Nat Cell Biol.

[2] Saxena S, Zou L (2022). Hallmarks of DNA replication stress. Mol Cell.

[3] Barker S, Weinfeld M, Murray D (2005). DNA–protein crosslinks: their induction, repair, and biological consequences. Mutat Res.

[4] Weickert P, Stingele J (2022). DNA–Protein Crosslinks and Their Resolution. Annu Rev Biochem.

[5] Novakova O, Kasparkova J, Malina J, Natile G, Brabec V (2003). DNA–protein cross-linking by trans-[PtCl 2 (E-iminoether) 2]. A concept for activation of the trans geometry in platinum antitumor complexes. Nucleic Acids Res.

[6] Chválová K, Brabec V, Kašpárková J (2007). Mechanism of the formation of DNA–protein cross-links by antitumor cisplatin. Nucleic Acids Res.

[7] Kuo HK, Griffith JD, Kreuzer KN (2007). 5-Azacytidine–Induced Methyltransferase-DNA Adducts Block DNA Replication In vivo. Cancer Res.

[8] Fu YV, Yardimci H, Long DT, Ho TV, Guainazzi A, Bermudez VP, Hurwitz J, van Oijen A, Schärer OD, Walter JC (2011). Selective Bypass of a Lagging Strand Roadblock by the Eukaryotic Replicative DNA Helicase. Cell.

[9] Nakano T, Miyamoto-Matsubara M, Shoulkamy MI, Salem AMH, Pack SP, Ishimi Y, Ide H (2013). Translocation and Stability of Replicative DNA Helicases upon Encountering DNA-Protein Cross-links*. J Biol Chem.

[10] Langston L, O’Donnell M (2017). Action of CMG with strand-specific DNA blocks supports an internal unwinding mode for the eukaryotic replicative helicase. eLife.

[11] Champoux JJ, Dulbecco R (1972). An Activity from Mammalian Cells That Untwists Superhelical DNA—A Possible Swivel For DNA Replication. Proc Natl Acad Sci USA.

[12] Kim RA, Wang JC (1989). Function of DNA topoisomerases as replication swivels in Saccharomyces cerevisiae. J Mol Biol.

[13] Bermejo R, Doksani Y, Capra T, Katou Y-M, Tanaka H, Shirahige K, Foiani M (2007). Top1- and Top2-mediated topological transitions at replication forks ensure fork progression and stability and prevent DNA damage checkpoint activation. Genes Dev.

[14] Pommier Y, Sun Y, Huang SN, Nitiss JL (2016). Roles of eukaryotic topoisomerases in transcription, replication and genomic stability. Nat Rev Mol Cell Biol.

[15] Been MD, Champoux JJ (1980). Breakage of single-stranded DNA by rat liver nicking-closing enzyme with the formation of a DNA-enzyme complex. Nucleic Acids Res.

[16] Champoux JJ (1981). DNA is linked to the rat liver DNA nicking-closing enzyme by a phosphodiester bond to tyrosine. J Biol Chem.

[17] Stewart L, Redinbo MR, Qiu X, Hol WGJ, Champoux JJ (1998). A Model for the Mechanism of Human Topoisomerase I. Science.

[18] Redinbo MR, Stewart L, Kuhn P, Champoux JJ, Hol WGJ (1998). Crystal Structures of Human Topoisomerase I in Covalent and Noncovalent Complexes with DNA. Science.

[19] Stivers JT, Harris TK, Mildvan AS (1997). Vaccinia DNA Topoisomerase I: Evidence Supporting a Free Rotation Mechanism for DNA Supercoil Relaxation †. Biochemistry.

[20] Koster DA, Croquette V, Dekker C, Shuman S, Dekker NH (2005). Friction and torque govern the relaxation of DNA supercoils by eukaryotic topoisomerase IB. Nature.

[21] Pommier Y, Barcelo JM, Rao VA, Sordet O, Jobson AG, Thibaut L, Miao Z, Seiler JA, Zhang H, Marchand C (2006). Repair of Topoisomerase I-Mediated DNA Damage. Prog Nucleic Acid Res Mol Biol.

[22] Stingele J, Schwarz MS, Bloemeke N, Wolf PG, Jentsch S (2014). A DNA-Dependent Protease Involved in DNA-Protein Crosslink Repair. Cell.

[23] Hsiang YH, Hertzberg R, Hecht S, Liu LF (1985). Camptothecin induces protein-linked DNA breaks via mammalian DNA topoisomerase I. J Biol Chem.

[24] Nitiss J, Wang JC (1988). DNA topoisomerase-targeting antitumor drugs can be studied in yeast. Proc Natl Acad Sci USA.

[25] Staker BL, Hjerrild K, Feese MD, Behnke CA, Burgin AB, Stewart L (2002). The mechanism of topoisomerase I poisoning by a camptothecin analog. Proc Natl Acad Sci USA.

[26] Staker BL, Feese MD, Cushman M, Pommier Y, Zembower D, Stewart L, Burgin AB (2005). Structures of Three Classes of Anticancer Agents Bound to the Human Topoisomerase I-DNA Covalent Complex. J Med Chem.

[27] Pommier Y (2009). DNA Topoisomerase I Inhibitors: Chemistry, Biology, and Interfacial Inhibition. Chem Rev.

[28] Hsiang YH, Lihou MG, Liu LF (1989). Arrest of replication forks by drug-stabilized topoisomerase I-DNA cleavable complexes as a mechanism of cell killing by camptothecin. Cancer Res.

[29] Holm C, Covey JM, Kerrigan D, Pommier Y (1989). Differential requirement of DNA replication for the cytotoxicity of DNA topoisomerase I and II inhibitors in Chinese hamster DC3F cells. Cancer Res.

[30] Pommier Y (2006). Topoisomerase I inhibitors: camptothecins and beyond. Nat Rev Cancer.

[31] Strumberg D, Pilon AA, Smith M, Hickey R, Malkas L, Pommier Y (2000). Conversion of Topoisomerase I Cleavage Complexes on the Leading Strand of Ribosomal DNA into 5′-Phosphorylated DNA Double-Strand Breaks by Replication Runoff. Mol Cell Biol.

[32] Chaudhuri AR, Hashimoto Y, Herrador R, Neelsen KJ, Fachinetti D, Bermejo R, Cocito A, Costanzo V, Lopes M (2012). Topoisomerase I poisoning results in PARP-mediated replication fork reversal. Nat Struct Mol Biol.

[33] Lin C-P, Ban Y, Lyu YL, Desai SD, Liu LF (2008). A Ubiquitin-Proteasome Pathway for the Repair of Topoisomerase I-DNA Covalent Complexes*. J Biol Chem.

[34] Lin C-P, Ban Y, Lyu YL, Liu LF (2009). Proteasome-dependent Processing of Topoisomerase I-DNA Adducts into DNA Double Strand Breaks at Arrested Replication Forks*. J Biol Chem.

[35] Baretic’ D, Jenkyn-Bedford M, Aria V, Cannone G, Skehel M, Yeeles JTP (2020). Cryo-EM Structure of the Fork Protection Complex Bound to CMG at a Replication Fork. Mol Cell.

[36] Calzada A, Hodgson B, Kanemaki M, Bueno A, Labib K (2005). Molecular anatomy and regulation of a stable replisome at a paused eukaryotic DNA replication fork. Genes Dev.

[37] Tourrière H, Versini G, Cordón-Preciado V, Alabert C, Pasero P (2005). Mrc1 and Tof1 Promote Replication Fork Progression and Recovery Independently of Rad53. Mol Cell.

[38] Hodgson B, Calzada A, Labib K (2007). Mrc1 and Tof1 Regulate DNA Replication Forks in Different Ways during Normal S Phase. Mol Biol Cell.

[39] Redon C, Pilch DR, Bonner WM (2006). Genetic Analysis of Saccharomyces cerevisiae H2A serine 129 mutant suggests a functional relationship between H2A and the sister-chromatid cohesion partners Csm3-Tof1 for the repair of topoisomerase I-induced DNA damage. Genetics.

[40] Hosono Y, Abe T, Higuchi M, Kajii K, Sakuraba S, Tada S, Enomoto T, Seki M (2014). Tipin Functions in the Protection against Topoisomerase I Inhibitor*. J Biol Chem.

[41] Leman AR, Noguchi C, Lee CY, Noguchi E (2010). Human Timeless and Tipin stabilize replication forks and facilitate sister-chromatid cohesion. J Cell Sci.

[42] Andersen AH, Gocke E, Bonven BJ, Nielsen OF, Westergaard O (1985). Topoisomerase I has a strong binding preference for a conserved hexadecameric sequence in the promotor region of the rRNA gene from Tetrahymena pyriformis. Nucleic Acids Res.

[43] Burgin AB, Huizenga BN, Nash HA (1995). A novel suicide substrate for DNA topoisomerases and site-specific recombinases. Nucleic Acids Res.

[44] Yeeles JTP, Deegan TD, Janska A, Early A, Diffley JFX (2015). Regulated eukaryotic DNA replication origin firing with purified proteins. Nature.

[45] Yeeles JTP, Janska A, Early A, Diffley JFX (2017). How the Eukaryotic Replisome Achieves Rapid and Efficient DNA Replication. Mol Cell.

[46] Taylor MRG, Yeeles JTP (2018). The Initial Response of a Eukaryotic Replisome to DNA Damage. Mol Cell.

[47] Devbhandari S, Jiang J, Kumar C, Whitehouse I, Remus D (2017). Chromatin Constrains the Initiation and Elongation of DNA Replication. Mol Cell.

[48] Vrtis KB, Dewar JM, Chistol G, Wu RA, Graham TGW, Walter JC (2021). Single-strand DNA breaks cause replisome disassembly. Mol Cell.

[49] Takai H, Aria V, Borges P, Yeeles JTP, de Lange T (2024). CST–polymerase α-primase solves a second telomere end-replication problem. Nature.

[50] Park H, Sternglanz R (1999). Identification and characterization of the genes for two topoisomerase I-interacting proteins from Saccharomyces cerevisiae. Yeast.

[51] Shyian M, Albert B, Zupan AM, Ivanitsa V, Charbonnet G, Dilg D, Shore D (2020). Fork pausing complex engages topoisomerases at the replisome. Genes Dev.

[52] Westhorpe R, Keszthelyi A, Minchell NE, Jones D, Baxter J (2020). Separable functions of Tof1/Timeless in intra-S-checkpoint signalling, replisome stability and DNA topological stress. Nucleic Acids Res.

[53] Safaric B, Chacin E, Scherr MJ, Rajappa L, Gebhardt C, Kurat CF, Cordes T, Duderstadt KE (2022). The fork protection complex recruits FACT to reorganize nucleosomes during replication. Nucleic Acids Res.

[54] Schalbetter SA, Mansoubi S, Chambers AL, Downs JA, Baxter J (2015). Fork rotation and DNA precatenation are restricted during DNA replication to prevent chromosomal instability. Proc Natl Acad Sci USA.

[55] Jones ML, Baris Y, Taylor MRG, Yeeles JTP (2021). Structure of a human replisome shows the organisation and interactions of a DNA replication machine. EMBO J.

[56] Svejstrup JQ, Christiansen K, Gromova II, Andersen AH, Westergaard O (1991). New technique for uncoupling the cleavage and religation reactions of Eukaryotic Topoisomerase I. The mode of action of camptothecin at a specific recognition site. J Mol Biol.

[57] Woo MH, Vance JR, Marcos ARO, Bailly C, Bjornsti M-A (2002). Active Site Mutations in DNA Topoisomerase I Distinguish the Cytotoxic Activities of Camptothecin and the Indolocarbazole, Rebeccamycin*. J Biol Chem.

[58] Somyajit K, Gupta R, Sedlackova H, Neelsen KJ, Ochs F, Rask M-B, Choudhary C, Lukas J (2017). Redox-sensitive alteration of replisome architecture safeguards genome integrity. Science.

[59] Coster G, Frigola J, Beuron F, Morris EP, Diffley JFX (2014). Origin Licensing Requires ATP Binding and Hydrolysis by the MCM Replicative Helicase. Mol Cell.

[60] On KF, Beuron F, Frith D, Snijders AP, Morris EP, Diffley JFX (2014). Prereplicative complexes assembled in vitro support origin-dependent and independent DNA replication. EMBO J.

[61] Frigola J, Remus D, Mehanna A, Diffley JFX (2013). ATPase-dependent quality control of DNA replication origin licensing. Nature.

[62] Hill J, Eickhoff P, Drury LS, Costa A, Diffley JFX (2020). The eukaryotic replisome requires an additional helicase to disarm dormant replication origins. bioRxiv.

[63] Guilliam TA, Yeeles JTP (2020). Reconstitution of translesion synthesis reveals a mechanism of eukaryotic DNA replication restart. Nat Struct Mol Biol.

[64] Jenkyn-Bedford M, Jones ML, Baris Y, Labib KPM, Cannone G, Yeeles JTP, Deegan TD (2021). A conserved mechanism for regulating replisome disassembly in eukaryotes. Nature.

[65] Punjani A, Rubinstein JL, Fleet DJ, Brubaker MA (2017). cryoSPARC: algorithms for rapid unsupervised cryo-EM structure determination. Nat Methods.

[66] Scheres SHW (2012). RELION: Implementation of a Bayesian approach to cryo-EM structure determination. J Struct Biol.

[67] Pettersen EF, Goddard TD, Huang CC, Couch GS, Greenblatt DM, Meng EC, Ferrin TE (2004). UCSF Chimera—A visualization system for exploratory research and analysis. J Comput Chem.

