# Supplementary material for: Mechanisms controlling replication fork stalling and collapse at topoisomerase 1 cleavage complexes
Source: Mol Cell. Author manuscript; Available in PMC 2024 Dec 9. (PMC7617106; doi:10.1016/j.molcel.2024.08.004)
Supplement: Document S2. Article plus supplemental information. [file EMS201616-supplement-Document_S2__Article_plus_supplemental_information_.pdf]

Molecular Cell, Volume *84*

Supplemental information

Mechanisms controlling replication fork stalling and collapse at topoisomerase 1 cleavage complexes

Rose Westhorpe, Johann J. Roske, and Joseph T.P. Yeeles

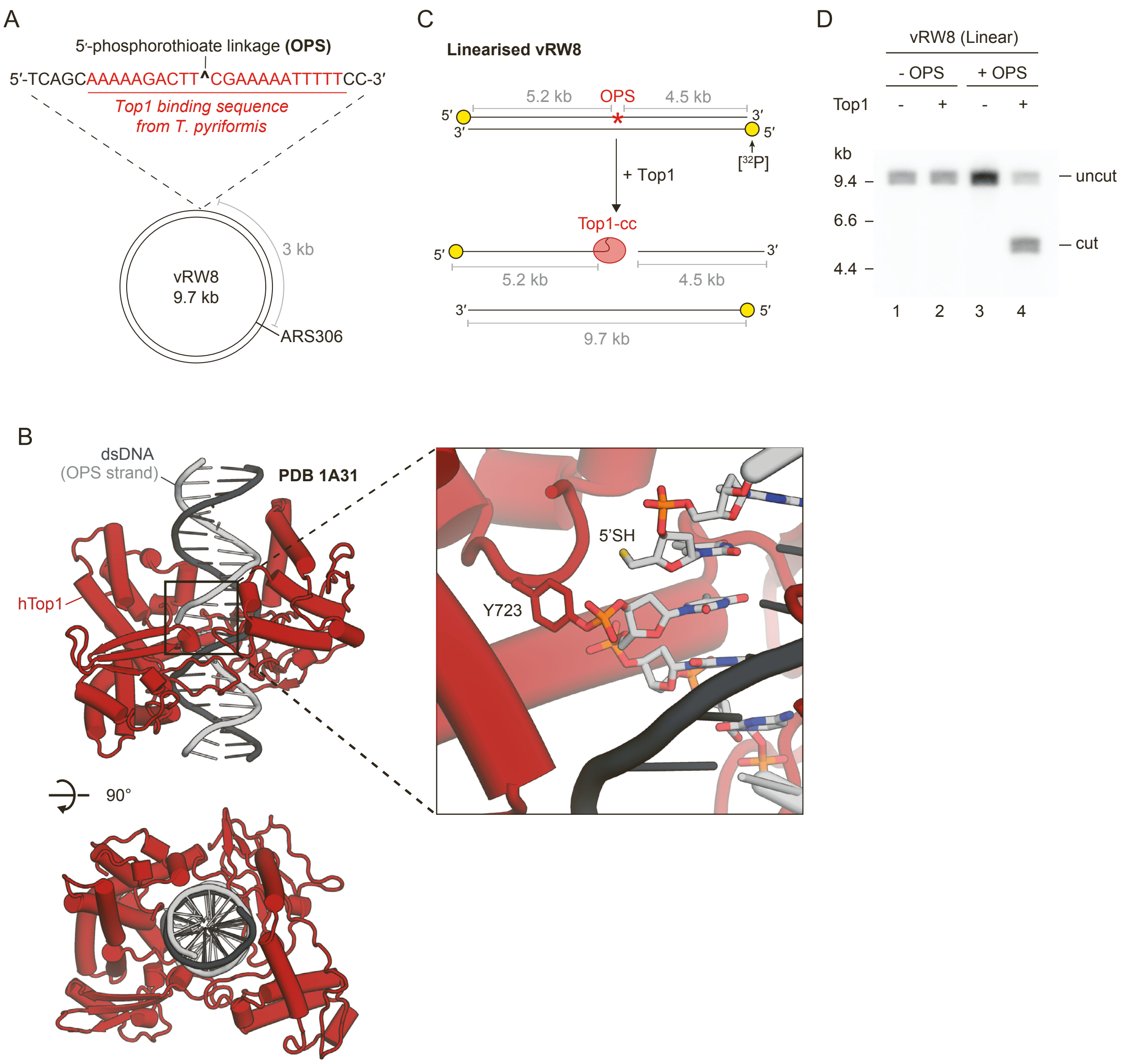

Figure S1. Generation of site-specific Top1 cleavage complexes in vitro, related to Figure 1.
(A) Schematic of the vector designed to site-specifically trap Top1-ccs.
(B) PDB deposition 1A31[S1]: Crystal structure of a reconstituted human Topoisomerase I cleavage complex using 5′ OPS-containing duplex DNA. A side view (top) and top view (bottom) of the complex demonstrate how the protein embraces both strands of the DNA duplex. Zoomed-in perspective (right) showing the active site tyrosine residue Y723 of Top1 in covalent complex with the DNA, with the 5′ sulfhydryl group that results from cleavage at the OPS site indicated.
(C) Schematic of the Top1-cleavage assay. Templates were linearised and [^{32}P]-end labelled prior to incubation with Top1. The expected products generated from Top1-cleavage are indicated.
(D) Denaturing agarose gel analysis of products generated in the Top1 cleavage assay outlined in (**C**).

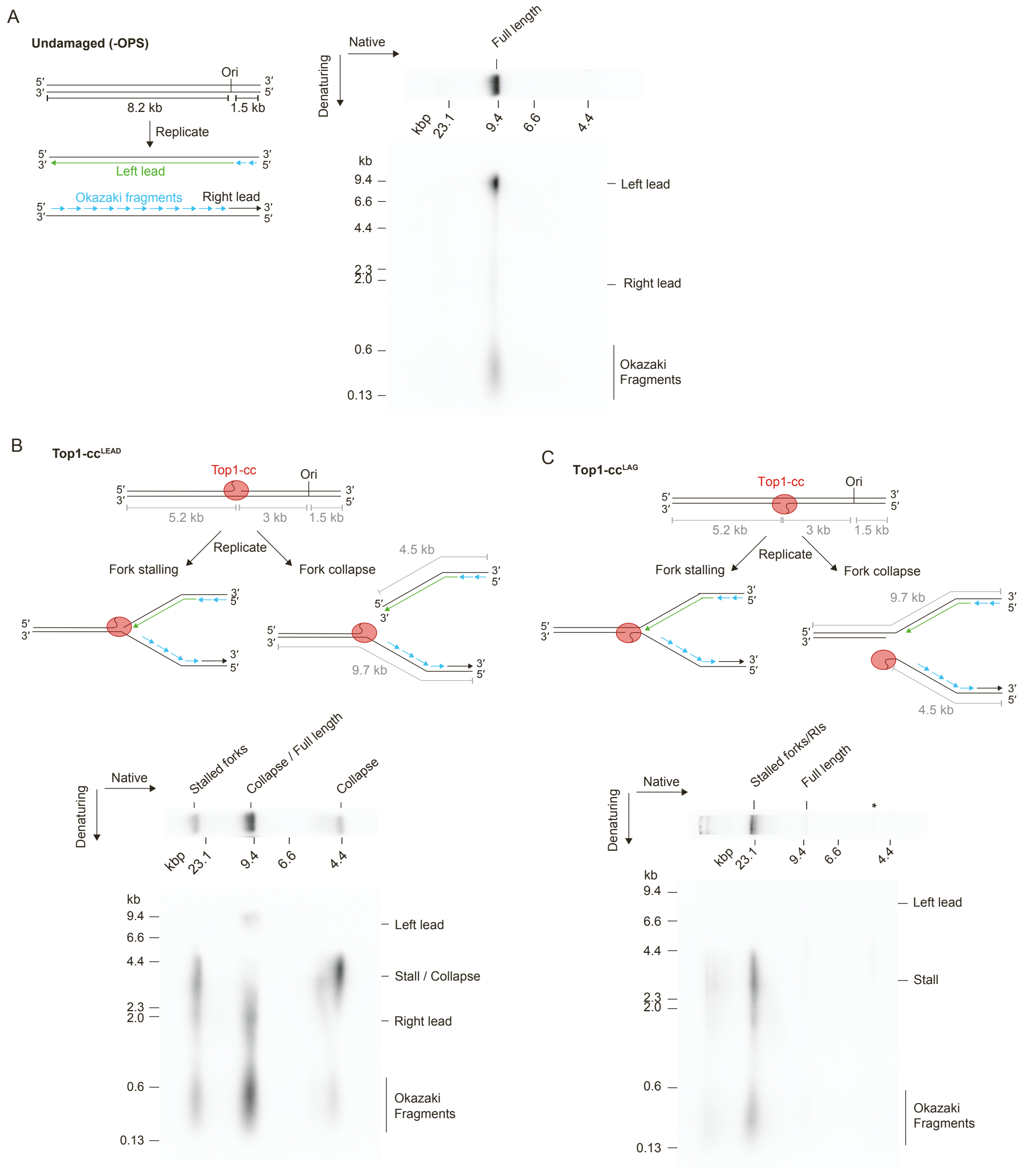

Figure S2. Two-dimensional (2D) analysis of replication products, related to Figure 1.
(A-C) Two-dimensional gel analysis of 30 min replication reactions performed on Undamaged (-OPS) (**A**), Top1-cc^{LEAD} (**B**) and Top1-cc^{LAG} (**C**) templates. The size and nascent strand composition of expected products is illustrated for each template. For all 2D gels, replication products were first run on native agarose gels before the gel slice containing these products was embedded into and run through a denaturing agarose gel. The asterisk in panel **C** indicates an ~5 kb product that is comprised mainly of leading-strand products and is therefore not the result of collapsed leftward moving replication forks. These products potentially arose due to low level origin-independent replication initiation occurring to the left of the Top1-cc.

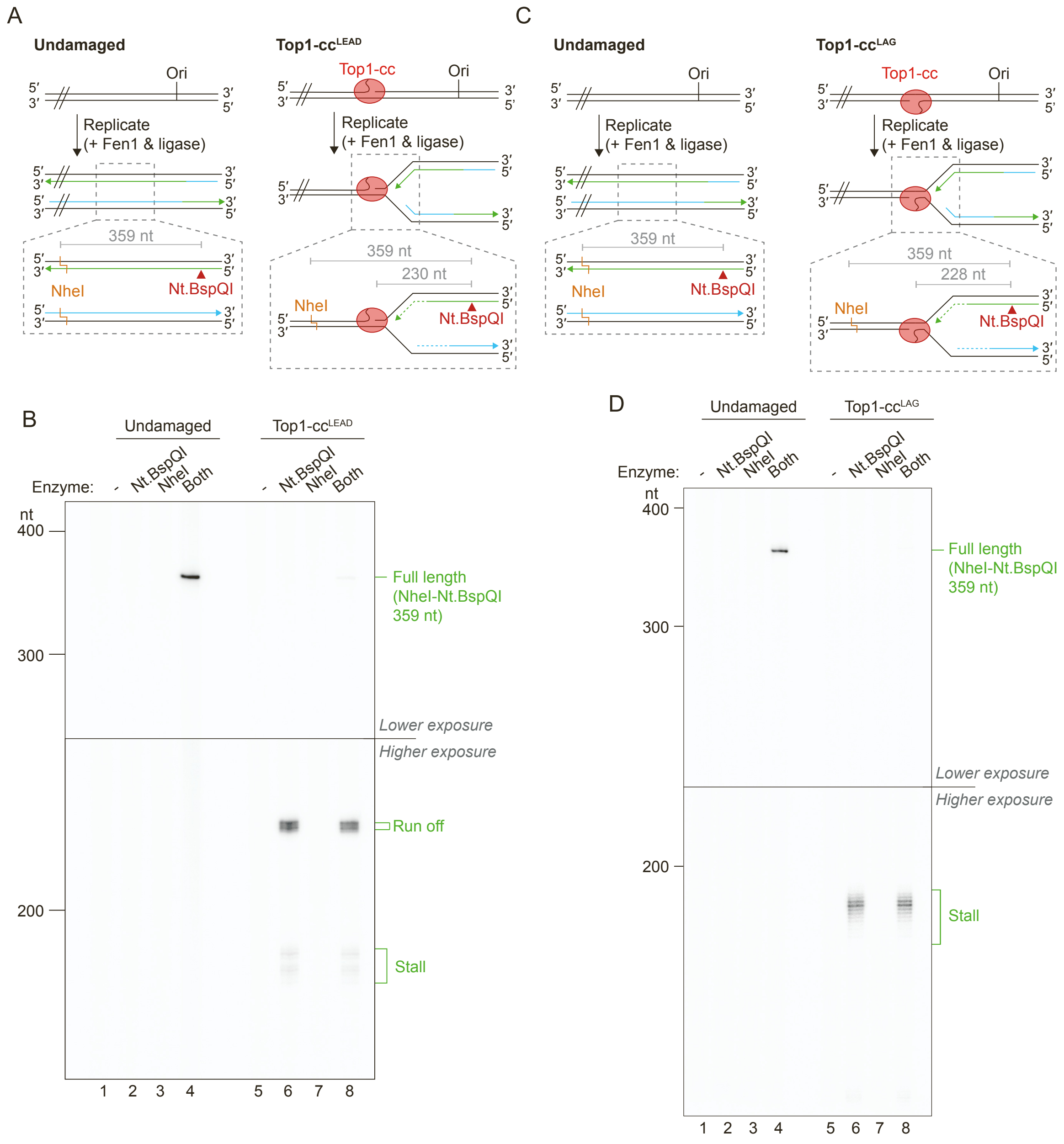

Figure S3. Analysis of stalled leading-strands at Top1-ccs, related to Figure 2

(A) Schematic of the post-reaction processing strategy used to liberate nascent leading strands from reactions on undamaged and Top1-cc^{LEAD} templates for denaturing polyacrylamide gel analysis. For clarity, the region of the template downstream of the dashed lines (//) is not shown.

(B) Denaturing polyacrylamide analysis gel of replication products (30 min reaction) generated on undamaged and Top1-cc^{LEAD} templates and digested post-replication with the indicated enzymes. Two exposures of the same gel are shown to aid visualisation of weaker bands.

(C) As in (**A**) for replication reactions performed on undamaged or Top1-cc^{LAG} templates.

(D) Experiment performed and analysed as in (**B**) but on undamaged and Top1-cc^{LAG} templates.

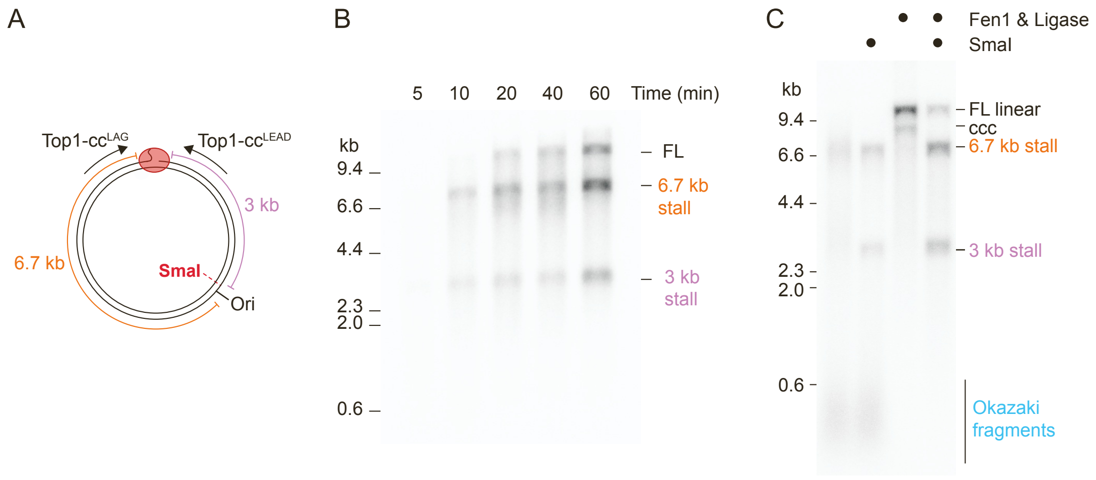

Figure S4. Replication products from circular Top1-cc-containing templates are digested into both stall and full-length nascent strands, related to Figure 4.

(**A**) Schematic of the circular template containing a single Top1-cc. The type of Top1-cc encountered by each replication fork is marked with arrows: the anticlockwise moving fork encounters a Top1-cc^{LEAD} ~ 3kb from the origin whilst the clockwise moving fork encounters a Top1-cc^{LAG} ~6.7 kb from the origin. The site of Sma1 cleavage is shown in red.

(**B**) Denaturing agarose gel analysis of products generated from a time course reaction on the template illustrated in (**A**) and digested with SmaI. FL = Full length. (**C**) Denaturing agarose gel analysis of products generated from a replication reaction on the template illustrated in (**A**). Replication reactions were conducted either in the presence or absence of Fen1 and Ligase to mature lagging strands and were quenched after 30 min. Samples were digested with SmaI after replication where indicated. FL = full length, ccc = covalently closed circular.

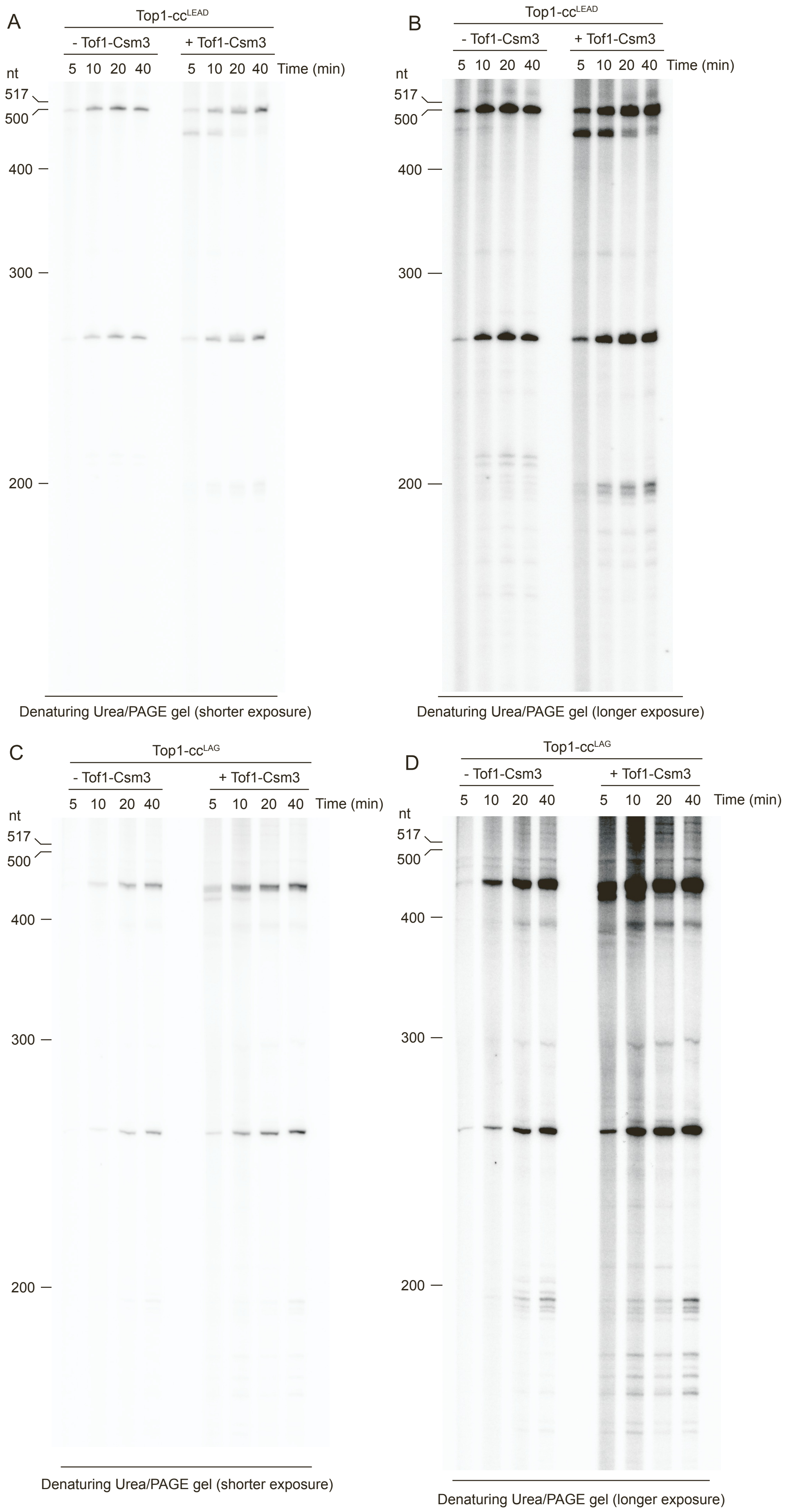

A
Top1-cc LEAD
- Tof1-Csm3
+ Tof1-Csm3
5 10 20 40 5 10 20 40 Time (min)
nt
517
500
400
300
200
Denaturing Urea/PAGE gel (shorter exposure)
B
Top1-cc LEAD
- Tof1-Csm3
+ Tof1-Csm3
5 10 20 40 5 10 20 40 Time (min)
nt
517
500
400
300
200
Denaturing Urea/PAGE gel (longer exposure)
C
Top1-cc LAG
- Tof1-Csm3
+ Tof1-Csm3
5 10 20 40 5 10 20 40 Time (min)
nt
517
500
400
300
200
Denaturing Urea/PAGE gel (shorter exposure)
D
Top1-cc LAG
- Tof1-Csm3
+ Tof1-Csm3
5 10 20 40 5 10 20 40 Time (min)
nt
517
500
400
300
200
Denaturing Urea/PAGE gel (longer exposure)

Figure S5. Full images of sequencing gels at both higher and lower exposures, related to Figure 5.
(A-B) Entire gel from Figure 5C shown at the lower **(A)** or higher **(B)** exposure.
(C-D) Entire gel from Figure 5D shown at the lower **(C)** or higher **(D)** exposure.

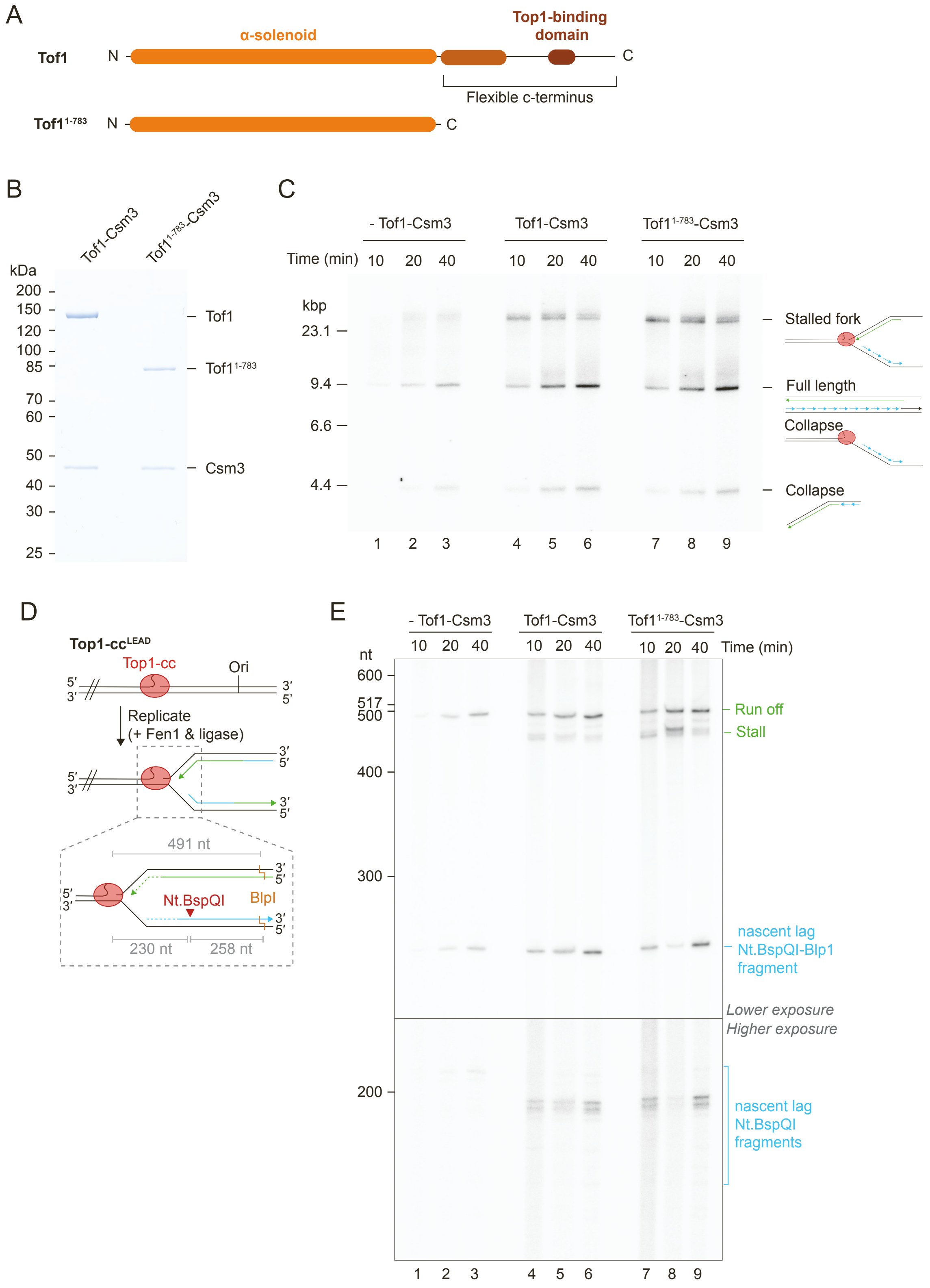

Figure S6. The C-terminal Top1-binding region of Tof1 is not required for replisome stalling at Top1-cc^{LEAD}, related to Figure 4.
(A) Schematic of wild type Tof1 and Tof1$^{1-783}$ showing basic domain architecture. The alpha-solenoid comprises residues 13-781[S2]. AlphaFold 2 predicts (accession AF-P53840-F1) that the flexible C-terminus of Tof1 contains two structured domains comprising residues 787-952 and 1058-1153, the latter of which has been described as the Top1-binding domain[S3-S5].

(B) Coomassie-stained 4-12% SDS-PAGE analysis of wild-type Tof1-Csm3 and $Tof1^{1\text{-}783}$-Csm3 complexes purified from budding yeast.
(C) Native agarose gel analysis of replication products generated in time course reactions on linear $Top1\text{-}cc^{LEAD}$ templates, without Tof1-Csm3, with wild-type Tof1-Csm3, or with $Tof1^{1\text{-}783}$-Csm3.
(D) Schematic of the post-reaction processing strategy used to liberate nascent leading and lagging strands from DNA replication reactions on the $Top1\text{-}cc^{LEAD}$ template for denaturing polyacrylamide gel analysis. Nascent leading strands are shown in green and matured lagging strands are shown in blue.
(E) Denaturing polyacrylamide gel analysis of replication products generated in time course reactions on a $Top1\text{-}cc^{LEAD}$ template, processed as indicated in (**A**). Two exposures of the same gel are shown to aid visualisation of weaker bands. Leading-strand products are labelled in green (Run off, Stall) and lagging-strand products are shown in blue (Nt.BspQI-Blp1 fragment, Nt.BspQI fragments). For clarity, the region of the template downstream of the dashed lines (//) is not shown.

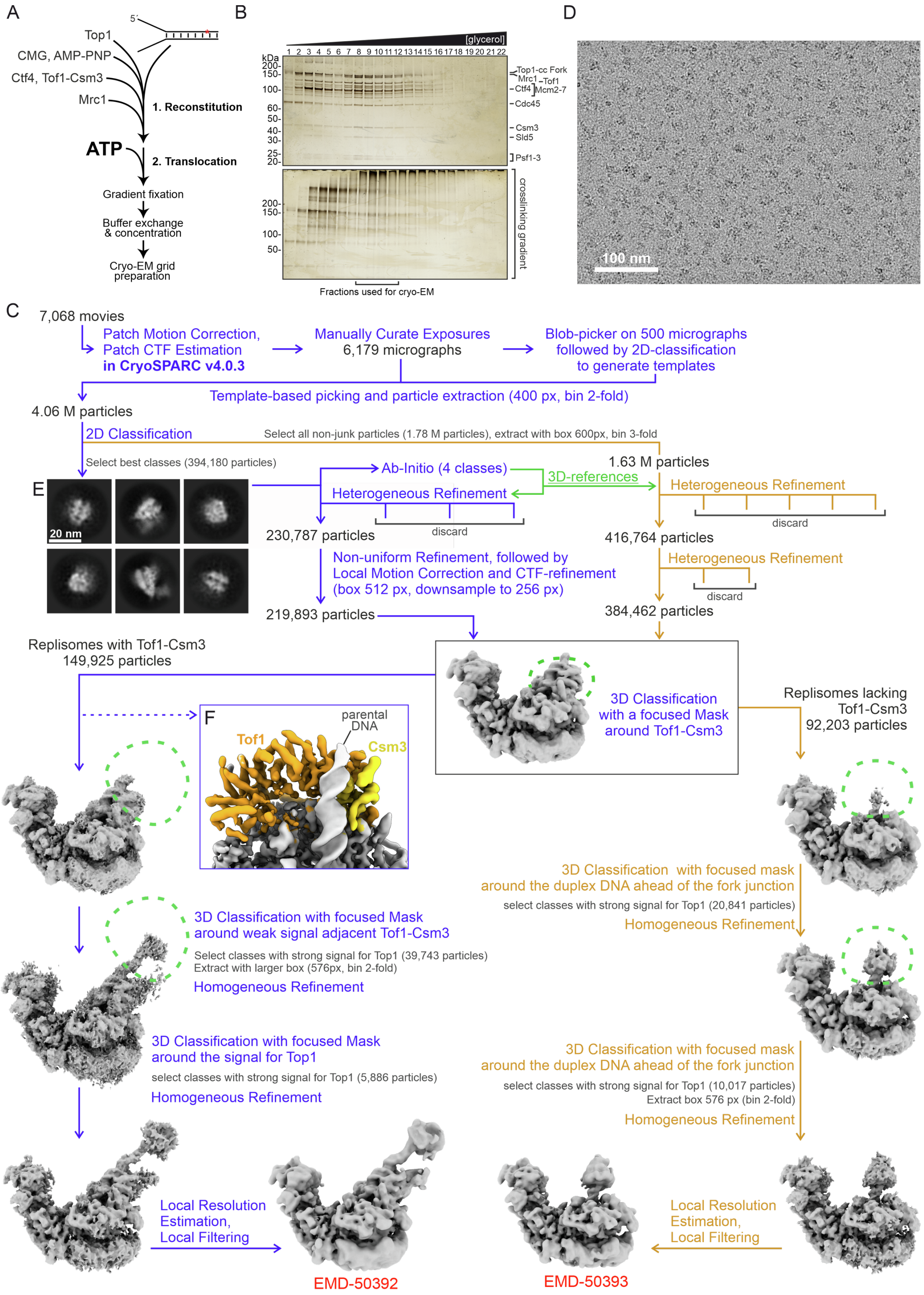
A
5'
Top1
CMG, AMP-PNP
Ctf4, Tof1-Csm3
Mrc1
1. Reconstitution
ATP
2. Translocation
Gradient fixation
Buffer exchange & concentration
Cryo-EM grid preparation
B
[glycerol]
kDa
Top1-cc Fork
Mrc1
Tof1
Ctf4
Mcm2-7
Cdc45
Csm3
Sld5
Psf1-3
crosslinking gradient
Fractions used for cryo-EM
D
100 nm
C
7,068 movies
Patch Motion Correction, Patch CTF Estimation in CryoSPARC v4.0.3
Manually Curate Exposures 6,179 micrographs
Blob-picker on 500 micrographs followed by 2D-classification to generate templates
Template-based picking and particle extraction (400 px, bin 2-fold)
4.06 M particles
2D Classification
Select all non-junk particles (1.78 M particles), extract with box 600px, bin 3-fold
Select best classes (394,180 particles)
1.63 M particles
E
20 nm
Ab-Initio (4 classes)
3D-references
Heterogeneous Refinement
Heterogeneous Refinement
discard
230,787 particles
discard
416,764 particles
Non-uniform Refinement, followed by Local Motion Correction and CTF-refinement (box 512 px, downsample to 256 px)
Heterogeneous Refinement
discard
219,893 particles
384,462 particles
Replisomes with Tof1-Csm3 149,925 particles
3D Classification with a focused Mask around Tof1-Csm3
Replisomes lacking Tof1-Csm3 92,203 particles
F
parental DNA
Tof1
Csm3
3D Classification with focused mask around the duplex DNA ahead of the fork junction
select classes with strong signal for Top1 (20,841 particles)
Homogeneous Refinement
3D Classification with focused Mask around weak signal adjacent Tof1-Csm3
Select classes with strong signal for Top1 (39,743 particles)
Extract with larger box (576px, bin 2-fold)
Homogeneous Refinement
3D Classification with focused Mask around the signal for Top1
select classes with strong signal for Top1 (5,886 particles)
Homogeneous Refinement
3D Classification with focused mask around the duplex DNA ahead of the fork junction
select classes with strong signal for Top1 (10,017 particles)
Extract box 576 px (bin 2-fold)
Homogeneous Refinement
Local Resolution Estimation, Local Filtering
EMD-50392
EMD-50393
Local Resolution Estimation, Local Filtering

Figure S7. Cryo-EM analysis of a budding yeast replisome stalled at a lagging-strand Top1-cc, related to Figure 6.

(**A**) Schematic outlining the *in vitro* reconstitution strategy used to generate complexes for cryo-EM. The model fork DNA is shown as a cartoon with a red asterisk indicating the bridging phosphorothioate modification.

(**B**) Silver-stained SDS-PAGE gels analysing 100 µl fractions taken across 10-30% glycerol gradients either in the absence (top) or presence (bottom) of crosslinking agents. Indicated fractions were used for cryo-EM sample preparation.

(**C**) Cryo-EM data processing pipeline. Red text denotes cryo-EM maps deposited in the EMDB.

(**D**) Representative cryo-EM micrograph obtained using a K3 direct electron detector (Gatan), after Patch CTF estimation and low-pass filtered at 3 Å. Scale bar, 100 nm.

(**E**) Representative 2D class averages with replisome-like features, obtained using 2D classification in cryoSPARC v4.

(**F**) Focussed view of the cryo-EM density for Tof1-Csm3 and the parental DNA duplex obtained via consensus refinement of replisome particles containing Tof1-Csm3.

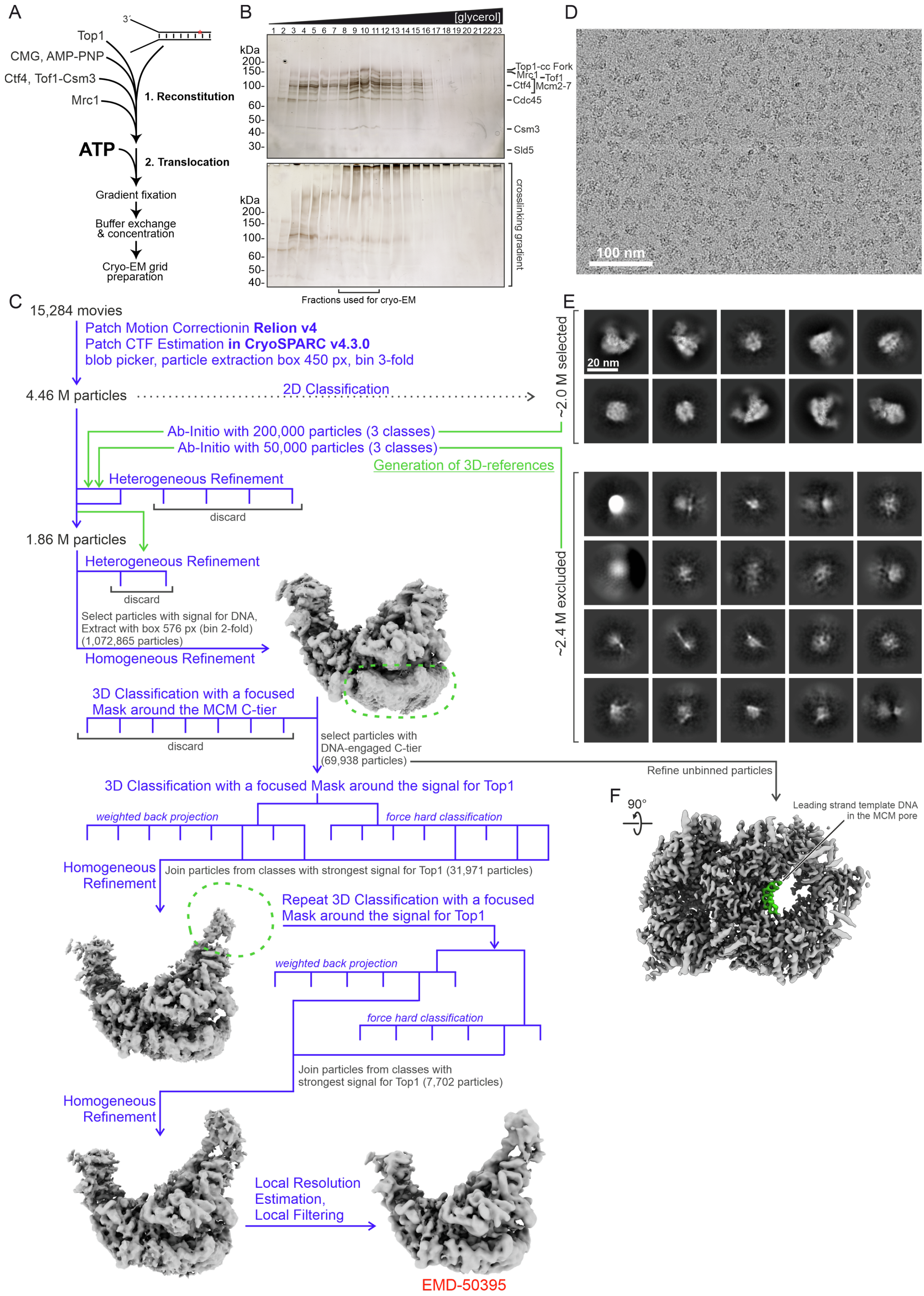

A
B
D
3´
Top1
CMG, AMP-PNP
Ctf4, Tof1-Csm3
Mrc1
1. Reconstitution
ATP
2. Translocation
Gradient fixation
Buffer exchange & concentration
Cryo-EM grid preparation
[glycerol]
kDa
Top1-cc Fork
Mrc1
Tof1
Ctf4
Mcm2-7
Cdc45
Csm3
Sld5
crosslinking gradient
Fractions used for cryo-EM
100 nm
C
15,284 movies
Patch Motion Correctionin Relion v4
Patch CTF Estimation in CryoSPARC v4.3.0
blob picker, particle extraction box 450 px, bin 3-fold
4.46 M particles
2D Classification
Ab-Initio with 200,000 particles (3 classes)
Ab-Initio with 50,000 particles (3 classes)
Generation of 3D-references
Heterogeneous Refinement
discard
1.86 M particles
Heterogeneous Refinement
discard
Select particles with signal for DNA, Extract with box 576 px (bin 2-fold) (1,072,865 particles)
Homogeneous Refinement
3D Classification with a focused Mask around the MCM C-tier
discard
select particles with DNA-engaged C-tier (69,938 particles)
Refine unbinned particles
3D Classification with a focused Mask around the signal for Top1
weighted back projection
force hard classification
Homogeneous Refinement
Join particles from classes with strongest signal for Top1 (31,971 particles)
Repeat 3D Classification with a focused Mask around the signal for Top1
weighted back projection
force hard classification
Join particles from classes with strongest signal for Top1 (7,702 particles)
Homogeneous Refinement
Local Resolution Estimation, Local Filtering
EMD-50395
E
~2.0 M selected
20 nm
~2.4 M excluded
F
90°
Leading strand template DNA in the MCM pore

Figure S8. Cryo-EM analysis of a budding yeast replisome stalled at a leading-strand Top1-cc, related to Figure 6.

(**A**) Schematic outlining the *in vitro* reconstitution strategy used to generate complexes for cryo-EM. The model fork DNA is shown as a cartoon with a red asterisk indicating the bridging phosphorothioate modification.

(**B**) Silver-stained SDS-PAGE gels analysing 100 µl fractions taken across 10-30% glycerol gradients either in the absence (top) or presence (bottom) of crosslinking agents. Indicated fractions were used for cryo-EM sample preparation.

(**C**) Cryo-EM data processing pipeline. Red text denotes the cryo-EM map deposited in the EMDB.

(**D**) Representative cryo-EM micrograph obtained using a K3 direct electron detector (Gatan), after Patch CTF estimation and low-pass filtered at 3 Å. Scale bar, 100 nm.

(**E**) Representative 2D class averages obtained using 2D classification in cryoSPARC v4 of all extracted particles.

(**F**) Rotated view on the consensus refinement of isolated particles with DNA-engaged MCM C-tier. Cryo-EM density for the bound leading-strand DNA is coloured green.

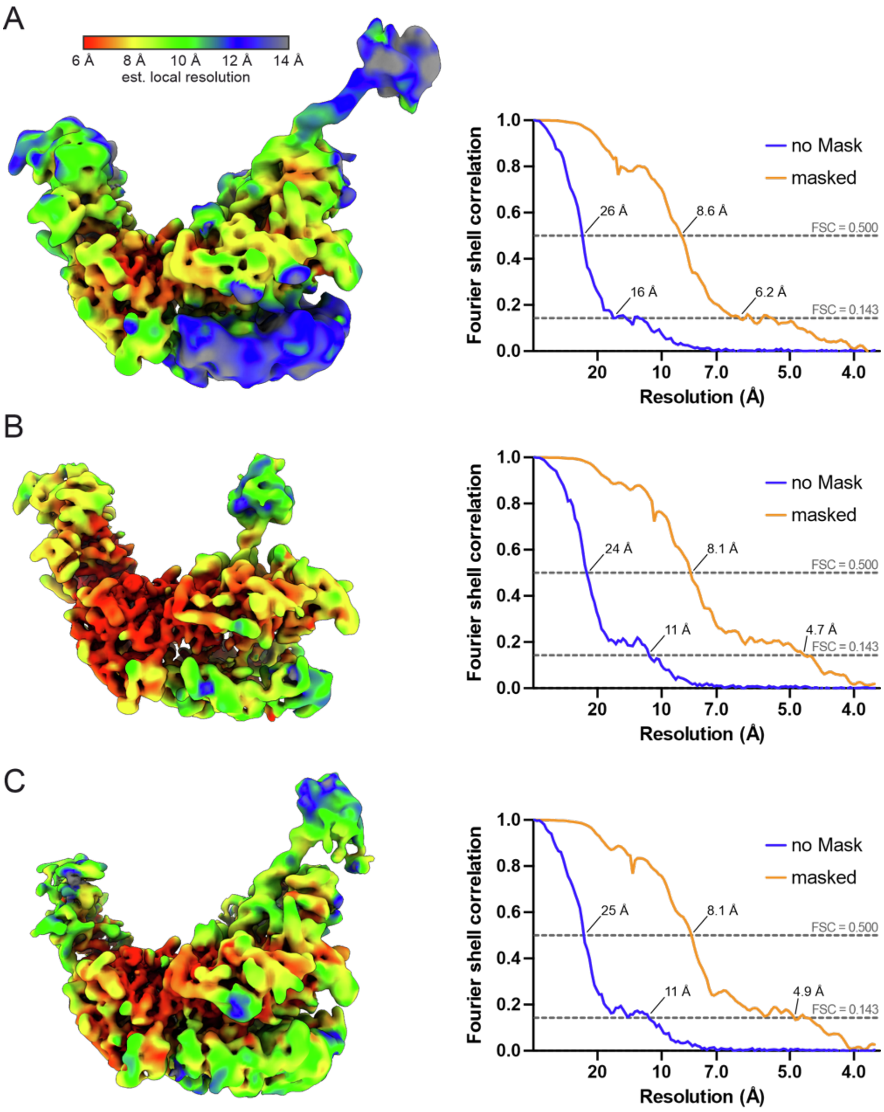

Figure S9. Local resolution and FSC for presented cryo-EM maps, related to Figure 6
(**A-C**) Left: Cryo-EM reconstructions of replisomes stalled at Top1-cc^{LAG} with (**A**) and without (**B**) density for Tof1-Csm3, or stalled at Top1-cc^{LEAD} (**C**). Maps were obtained via consensus refinement followed by local filtering and are coloured by local resolution according to inset key in panel (**A**), calculated using cryoSPARC. Adjacent graphs display the Fourier shell correlation of the refined maps. Resolutions are indicated for FSC=0.5 and FSC=0.143 cut-off values, calculated in presence or absence of a mask.

Plasmid name	Source	Notes/details
**ZN5_BspQI_Rem**	This study	Derivative of ZN5[S6] with Nt.BspQI nicking site removed. Base template used for generation of vRW8.
**vRW8**	This study	Vector containing a Top1-binding sequence and Nt.BbvCI nicking sites to insert OPS-modified oligos. Generated by insertion of duplexed oligos "ZN3_Top1_3_Top" and "ZN3_Top1_3_Bot" into the BamHI and PstI sites of vector ZN5_BspQI_Rem
**vRW11**	This study	Nt.BspQI site inserted into bottom strand 230 bp upstream of Top1-cc forming site in vRW8. Used for mapping nascent leading-strand synthesis in replication reactions. Generated by PCR mutagenesis of vRW8 with primers oRW40 and oRW41.
**vRW12**	This study	Nt.BspQI site inserted into top strand 230 bp upstream of Top1-cc forming site in vRW8. Used for mapping nascent strand synthesis in replication reactions. Generated by PCR mutagenesis of vRW8 with primers oRW42 and oRW43.
**vRW23**	This study	Top1 binding sequence inverted in vRW11 such that oRW30 is ligated into the bottom strand of the template and forms a Top1-cc on this strand. Generated by insertion of duplexed oligos oRW92 and oRW93 into the BamHI and PstI sites of vector vRW11.
**vRW24**	This study	Top1 binding sequence inverted in vRW12 such that oRW30 is ligated into the bottom strand of the template and forms a Top1-cc on this strand. Generated by insertion of duplexed oligos oRW92 and oRW93 into the BamHI and PstI sites of vector vRW12.
**vRW19**	This study	Nt.BspQI site inserted into bottom strand 300 bp downstream of Top1-cc forming site in vRW8. Generated by PCR mutagenesis of vRW8 with primers oRW78 and oRW79.

Table S1: Plasmids used and constructed for this study

Oligo name	Sequence (5'-3')	Used for/Notes
**ZN3_Top1_3_Top**	GATCCGCAGTACCAACCTCAGCAAAAAGACTTCGAAAAATTTTTCCTCAGCGCAGTACCAACTGCA	Duplexed with ZN3_Top1_3_Bot and inserted into BamHI/PstI sites of ZN5_BspQI_Rem to generate vRW8
**ZN3_Top1_3_Bot**	GTTGGTACTGCGCTGAGGAAAAATTTTTCGAAGTCTTTTTGCTGAGGTTGGTACTGCG	Duplexed with ZN3_Top1_3_Top and inserted into BamHI/PstI sites of ZN5_BspQI_Rem to generate vRW8
**oRW25**	GTTGGTACTGCGCTGAGGAAAAATTTTTCGAAGTCTTTTTGCTGAGGTTGGTACTGCG	Competitor oligo used in generation of OPS-containing templates (Top1-cc^{LEAD} templates)
**oRW97**	GCAGTACCAAGCTGAGGAAAAATTTTTCGAAGTCTTTTTGCTGAGGGCAGTACCAAC	Competitor oligo used in generation of OPS-containing templates (Top1-cc^{LAG} templates)
**oRW30**	TCAGCAAAAAGACTT^CGAAAAATTTTTCC	Oligo containing internal 5'-bridging phosphorothioate modification. Inserted into Nt.BbvCI sites of templates used for in vitro DNA replication reactions. Also used in construction of leading- and lagging-strand arms of model forks used in cryo-EM experiments.
**oRW40**	AGAAGAGCACGACATCCAAAATCTTTTTTCC	Mutagenesis of vRW8 to insert Nt.BspQI nicking site for mapping experiments
**oRW41**	ATCCAAATATTTTAAGAGCAACACA	Mutagenesis of vRW8 to insert Nt.BspQI nicking site for mapping experiments
**oRW42**	GCTCTTCGATTTGGATACGACATCCAAAAT	Mutagenesis of vRW8 to insert Nt.BspQI nicking site for mapping experiments
**oRW43**	ATTTTAAGAGCAACACATACCTTAC	Mutagenesis of vRW8 to insert Nt.BspQI nicking site for mapping experiments
**oRW78**	GCTCTTCCCATCCAATGAGTACGGAAGACCG	Mutagenesis of vRW8 to insert Nt.BspQI nicking site for mapping experiments
**oRW79**	TGCTGTTGACCATTCTGTTGCG	Mutagenesis of vRW8 to insert Nt.BspQI nicking site for mapping experiments
**oRW92**	GATCCGCAGTACCAAGCTGAGGAAAAATTTTTCGAAGTCTTTTTGCTGAGGGCAGTACCAACTGCA	Top of duplex for inserting Top1-cc sequence in reverse orientation into BamHI/PstI sites of vectors
**oRW93**	GTTGGTACTGCCCTCAGCAAAAAGACTTCGAAAAATTTTTCCTCAGCTTGGTACTGCG	Bottom of duplex for inserting Top1-cc sequence in reverse orientation into BamHI/PstI sites of vectors
**oJR016**	GGCAGGCAGGCAGGCAGGCAGGCAGGCAGGCAGGCAGGCCACACTCTCCAATTCTCTAATCACTTACCATCACTTCCTACTCTATGGTTTATTGACAAGGAAAAATTTTTCGAAGTCTTTTTGCTGATTACTTATACACTGTTACAT	Lagging strand of Top1-cc^{LEAD} fork used in cryo-EM experiments

**oJR010**	ATGTAACAGTGTATAAGTAA	Used in construction of an OPS-containing leading strand for the Top1-cc^{LEAD} fork used in cryo-EM experiments (with oJR11, oJR12 and oRW30)
**oJR015**	TGCTGATTACTTATA	1 of 3 splint oligos used for ligation of oligos into a leading strand of Top1-cc^{LEAD} fork for cryo-EM experiments (1/3)
**oJR014**	ATTGACAAGGAAAAATTTTT	1 of 3 splint oligos used for ligation of oligos into a leading strand of Top1-cc^{LEAD} fork for cryo-EM experiments (2/3)
**oJR011**	TTGTCAATAAACCATAGAGTAG<br>GAAGTGATGGTAAGTGATTAG<br>A	Used in construction of an OPS-containing leading strand for the Top1-cc^{LEAD} fork used in cryo-EM experiments (with oJR10, oJR12 and oRW30)
**oJR013**	CCAATTCTCTAATCA	1 of 3 splint oligos used for ligation of oligos into a leading strand of Top1-cc^{LEAD} fork for cryo-EM experiments (3/3)
**oRJ012**	GAATTGGAGAGTGTGTTTTTTTT<br>TTTTTTTTTTTTTTTTTTTTTTTT<br>TTTTCGATAGGCCGATAGATTT<br>TTTTTTTTTTTTTTTTTTTTTTTT<br>TTTTTTTT	Used in construction of an OPS-containing leading strand for the Top1-cc^{LEAD} fork used in cryo-EM experiments (with oJR10, oJR11 and oRW30)
**oRW74**	ATGTAACAGTGTATAAGTAAGG<br>AAAAATTTTTCGAAGTCTTTTTG<br>CTGATTGTCAATAAACCATAGA<br>GTAGGAAGTGATGGTAAGTGA<br>TTAGAGAATTGGAGAGTGTGTT<br>TTTTTTTTTTTTTTTTTTTTTTTT<br>TTTTTTTTTTTTTT	Leading strand of Top1-cc^{LAG} fork used in cryo-EM experiments
**oRW76**	TTACTTATACACTGTTACAT	Used in construction of an OPS-containing lagging strand for the Top1-cc^{LAG} fork used in cryo-EM experiments (with oRW75 and oRW30)
**oRW75**	GGCAGGCAGGCAGGCAGGCA<br>GGCAGGCAGGCAGGCAGGCA<br>CACACTCTCCAATTCTCTAATC<br>ACTTACCATCACTTCCTACTCT<br>ATGGTTTATTGACAA	Used in construction of an OPS-containing lagging strand for the Top1-cc^{LAG} fork used in cryo-EM experiments (with oRW76 and oRW30)
**oJR001**	GTATAAGTAAGGAAAAA	1 of 2 splint oligos used for ligation of oligos into a lagging- strand of Top1-cc^{LAG} fork for cryo-EM experiments (1/2)
**oJR002**	GCTGATTGTCAAT	1 of 2 splint oligos used for ligation of oligos into a lagging- strand of Top1-cc^{LAG} fork for cryo-EM experiments (2/2)

Table S2: Oligonucleotides used in this study

Protein	Affinity Tag	Purified as in	Purification Steps	Final storage buffer
**Cdt1-Mcm2-7**	N-terminal cleavable CBP tag on Mcm3	Coster et al., 2014[S7].	1. Calmodulin-Sepharose 4B<br>2. Superdex 200	45 mM HEPES-KOH pH 7.6, 100 mM KOAc, 5 mM MgOAc, 0.02% NP40, 10% glycerol
**Cdc6 (Expressed in *E. coli*)**	N-terminal cleavable GST tag	Coster et al., 2014[S7].	1. Glutathione Sepharose 4B<br>2. Bio-Gel HT Hydroxyapatite	50 mM K2HPO4/KH2PO4 pH 7.5, 5 mM MgCl2, 1% Triton X-100, 1 mM DTT
**DDK**	CBP tag on Dbf4	On et al., 2014[S8].	1. Calmodulin-Sepharose 4B<br>2. Lambda phosphatase dephosphorylation<br>3. Superdex 200	25 mM Hepes-KOH pH 7.6, 0.1 mM EGTA, 0.1 mM EDTA, 0.02% NP-40, 10% glycerol, 100 mM K-Glutamate, 2 mM β-mercaptoethanol
**ORC**	Cleavable CBP tag on Orc1	Frigola et al., 2013[S9].	1. Calmodulin-Sepharose 4B<br>2. Superdex 200	25 mM HEPES-KOH pH 7.6, 0.05% NP-40 and 10% glycerol, 300 mM KOAc
**S-CDK (*clbΔ1-100*[10])**	Cleavable CPB tag on Clb5	Yeeles et al., 2015[S10] and Hill et al., 2020[S11].	1. Calmodulin Sepharose 4B<br>2. TEV cleavage (elution)<br>3. Superose 6	40 mM HEPES-KOH pH 7.6, 10% glycerol, 0.02% v/v NP-40-S, 300 mM KOAc
**Dpb11**	C-terminal 3xFLAG tag	Yeeles et al., 2015[S10].	1. Anti-FLAG M2 Agarose<br>2. MonoS	25 mM HEPES-KOH pH 7.6, 10% glycerol, 0.02% (v/v) NP-40-S, 1 mM EDTA, 1 mM DTT, 300mM KOAc
**GINS (Expressed in *E. coli*)**	N-terminal His tag on Psf3	Yeeles et al., 2015[S10].	1. Ni-NTA Agarose<br>2. MonoQ<br>3. Superdex 200	25 mM HEPES-KOH pH 7.6, 10% glycerol, 0.02% (v/v) NP-40-S, 1 mM EDTA, 1 mM DTT, 200mM KOAc
**Cdc45**	Internal 2xFLAG tag	Yeeles et al., 2015[S10].	1. Anti-FLAG M2 Agarose<br>2. Bio-Gel HT Hydroxyapatite	25 mM HEPES-KOH pH 7.6, 10% glycerol, 1 mM EDTA, 1 mM DTT, 300mM KOAc
**Mcm10 (Expressed in *E. coli*)**	N-terminal His tag	Yeeles et al., 2015[S10].	1. Ni-NTA Agarose<br>2. MonoS (2x)	25 mM HEPES-KOH pH 7.6, 10% glycerol, 0.01% (v/v) NP-40-S, 1 mM EDTA, 1 mM DTT, 200 mM K-glutamate
**Polymerase ε**	C-terminal CBP tag on Dpb4	Yeeles et al., 2015[S10].	1. Calmodulin-Sepharose 4B<br>2. HiTrap Heparin HP<br>3. Superdex 200	25 mM HEPES-KOH pH 7.6, 10% glycerol, 1 mM DTT, 500 mM KOAc
**Ctf4**	N-terminal CBP tag	Yeeles et al., 2015[S10].	1. Calmodulin-Sepharose 4B<br>2. MonoQ<br>3. Superdex 200	25 mM Tris-HCl pH 7.2, 10% glycerol, 1 mM DTT, 75 mM NaCl, 1 mM EDTA
**RPA**	Untagged	Baretić et al., 2020[S2].	1. Nucleic acid precipitation with	25 mM Tris HCl pH 7.5, 1 mM EDTA, 10 %

			Polymin P<br>2. Ammonium sulfate precipitation<br>3. HiTrap Blue HP<br>4. ssDNA Cellulose<br>5. MonoQ	glycerol, 100 mM NaCl, 1 mM DTT
**RFC**	N-terminal CBP tag on Rfc3	Yeeles et al., 2017[S12].	1. Calmodulin-Sepharose 4B<br>2. MonoS<br>3. Superdex 200	25 mM HEPES-KOH pH 7.6, 10% glycerol, 1 mM DTT, 1 mM EDTA, 150 mM NaCl
**PCNA (Expressed in *E. coli*)**	Untagged	Yeeles et al., 2017[S12].	1. Nucleic acid precipitation with Polymin P<br>2. Ammonium sulfate precipitation<br>3. HiTrap SP HP (flow through)<br>4. HiTrap Heparin HP (flow through)<br>5. HiTrap DEAE Fast Flow<br>6. MonoQ<br>7. Superdex 200	25 mM Tris-HCl pH 7.2, 10% glycerol, 1 mM EDTA, 150 mM NaCl.
**Tof1-Csm3**	Cleavable N-terminal CBP tag on Csm3	Baretić et al., 2020[S2].	1. Calmodulin-Sepharose 5B<br>2. TEV cleavage/elution<br>3. MonoQ<br>4. Superdex 200	25 mM Tris-HCl pH 7.2, 10 % (v/v) glycerol, 0.02% (v/v) NP-40-S, 1mM DTT, 150 mM NaCl
**Tof1$^{1-783}$-Csm3**	Cleavable N-terminal CBP tag on Csm3	Baretić et al., 2020[S2].	1. Calmodulin-Sepharose 5B<br>2. TEV cleavage/elution<br>3. MonoQ<br>4. Superdex 200	25 mM Tris-HCl pH 7.2, 10 % (v/v) glycerol, 0.02% (v/v) NP-40-S, 1mM DTT, 150 mM NaCl
**Polymerase α-primase**	N-terminal CBP tag on Pri1	Yeeles et al., 2017[S12].	1. Calmodulin-Sepharose 4B<br>2. MonoQ<br>3. Superdex 200	25mM HEPES-KOH pH 7.6, 10% (v/v) glycerol, 400 mM KOAc, 0.02% (v/v) NP-40-S, 1mM DTT, 1 mM EDTA
**Polymerase δ**	C-terminal CBP tag on Pol32	Yeeles et al., 2017[S12].	1. Calmodulin-Sepharose 4B<br>2. HiTrap Heparin HP<br>3. Superdex 200	25 mM Tris-HCl pH 7.2, 10% glycerol, 0.02% NP-40-S, 1 mM EDTA, 1 mM DTT, 150 mM NaCl
**Mrc1**	C-terminal 2xFLAG tag	Baretić et al., 2020[S2].	1. Anti-FLAG M2 Agarose<br>2. Superose 6	25 mM Tris-HCl pH 7.2, 10 % (v/v) glycerol, 0.02% (v/v) NP-40-S, 1mM DTT, 150 mM NaCl
**Sld3/7**	Cleavable C-terminal TCP tag	Yeeles et al., 2015[S10].	1. IgG Sepharose Fast Flow<br>2. TEV removal with Ni-NTA Agarose<br>3. Superdex 200	25 mM HEPES-KOH pH 7.6, 10% glycerol, 0.02% (v/v) NP-40-S, 1 mM EDTA, 1 mM DTT, 500 mM KCl
**Sld2**	C-terminal 3xFLAG tag	Yeeles et al., 2015[S10].	1. Ammonium sulfate precipitation<br>2. Anti-FLAG M2 Agarose	25 mM HEPES-KOH pH 7.6, 0.02% (v/v) NP-40-S, 1 mM EDTA,

			3. HiTrap SP HP	1 mM DTT 40% (v/v) glycerol, 350 mM KCl
**Fen1**	C-terminal 3xFLAG tag	Guilliam and Yeeles, 2020[S13].	1. Anti-FLAG M2 Agarose<br>2. HiTrap Heparin HP	25 mM Tris-HCl pH 7.5, 0.02% (v/v) NP-40-S, 1 mM DTT, 1 mM EDTA, 10% (v/v) glycerol, 200mM NaCl
**Cdc9 (Ligase)**	C-terminal 2xFLAG tag	Guilliam and Yeeles, 2020[S13].	1. Anti-FLAG M2 Agarose<br>2. MonoQ	25mM HEPES-KOH pH 7.6, 10% (v/v) glycerol, 200 mM KOAc, 0.02% (v/v) NP-40-S, 1mM DTT
**Top1**	Cleavable N-terminal CBP tag	Yeeles et al., 2015[S10] (With different storage buffer).	1. Calmodulin-Sepharose 4B<br>2. TEV removal with Talon column<br>3. Superdex 200	Different storage buffer: 25mM HEPES-KOH pH 7.6, 10% (v/v) glycerol, 400 mM KOAc, 0.02% (v/v) NP-40-S, 1mM DTT
**CMG**	2xFLAG internal tag on Cdc45<br>N-terminal CBP cleavable tag on Mcm3<br>N-terminal His tag on Psf3	Baretić et al., 2020[S2].	1. Anti-FLAG M2 Agarose<br>2. Calmodulin-Sepharose 4B<br>3. MonoQ	25 mM HEPES-KOH pH 7.6, 40 mM KOAc, 40 mM K-glutamate, 2 mM Mg(OAc)2, 0.25 mM EDTA, 0.5 mM TCEP, 20% glycerol

Table S3: Summary of purification strategies for proteins used in this study

Supplementary Reference List

S1. Redinbo, M.R., Stewart, L., Kuhn, P., Champoux, J.J., and Hol, W.G.J. (1998). Crystal Structures of Human Topoisomerase I in Covalent and Noncovalent Complexes with DNA. Science 279, 1504–1513. https://doi.org/10.1126/science.279.5356.1504.

S2. Baretić, D., Jenkyn-Bedford, M., Aria, V., Cannone, G., Skehel, M., and Yeeles, J.T.P. (2020). Cryo-EM Structure of the Fork Protection Complex Bound to CMG at a Replication Fork. Mol. Cell 78, 926-940.e13. https://doi.org/10.1016/j.molcel.2020.04.012.

S3. Shyian, M., Albert, B., Zupan, A.M., Ivanitsa, V., Charbonnet, G., Dilg, D., and Shore, D. (2020). Fork pausing complex engages topoisomerases at the replisome. Genes Dev. 34, 87–98. https://doi.org/10.1101/gad.331868.119.

S4. Westhorpe, R., Keszthelyi, A., Minchell, N.E., Jones, D., and Baxter, J. (2020). Separable functions of Tof1/Timeless in intra-S-checkpoint signalling, replisome stability and DNA topological stress. Nucleic Acids Res. 48, gkaa963-. https://doi.org/10.1093/nar/gkaa963.

S5. Safaric, B., Chacin, E., Scherr, M.J., Rajappa, L., Gebhardt, C., Kurat, C.F., Cordes, T., and Duderstadt, K.E. (2022). The fork protection complex recruits FACT to reorganize nucleosomes during replication. Nucleic Acids Res. 50, 1317–1334. https://doi.org/10.1093/nar/gkac005.

S6. Taylor, M.R.G., and Yeeles, J.T.P. (2018). The Initial Response of a Eukaryotic Replisome to DNA Damage. Mol Cell 70, 1067-1080.e12. https://doi.org/10.1016/j.molcel.2018.04.022.

S7. Coster, G., Frigola, J., Beuron, F., Morris, E.P., and Diffley, J.F.X. (2014). Origin Licensing Requires ATP Binding and Hydrolysis by the MCM Replicative Helicase. Mol. Cell 55, 666–677. https://doi.org/10.1016/j.molcel.2014.06.034.

S8. On, K.F., Beuron, F., Frith, D., Snijders, A.P., Morris, E.P., and Diffley, J.F.X. (2014). Prereplicative complexes assembled in vitro support origin‐dependent and independent DNA replication. EMBO J. 33, 605–620. https://doi.org/10.1002/embj.201387369.

S9. Frigola, J., Remus, D., Mehanna, A., and Diffley, J.F.X. (2013). ATPase-dependent quality control of DNA replication origin licensing. Nature 495, 339–343. https://doi.org/10.1038/nature11920.

S10. Yeeles, J.T.P., Deegan, T.D., Janska, A., Early, A., and Diffley, J.F.X. (2015). Regulated eukaryotic DNA replication origin firing with purified proteins. Nature 519, 431–435. https://doi.org/10.1038/nature14285.

S11. Hill, J., Eickhoff, P., Drury, L.S., Costa, A., and Diffley, J.F.X. (2020). The eukaryotic replisome requires an additional helicase to disarm dormant replication origins. bioRxiv, 2020.09.17.301366. https://doi.org/10.1101/2020.09.17.301366.

S12. Yeeles, J.T.P., Janska, A., Early, A., and Diffley, J.F.X. (2017). How the Eukaryotic Replisome Achieves Rapid and Efficient DNA Replication. Mol. Cell 65, 105–116. https://doi.org/10.1016/j.molcel.2016.11.017.

S13. Guilliam, T.A., and Yeeles, J.T.P. (2020). Reconstitution of translesion synthesis reveals a mechanism of eukaryotic DNA replication restart. Nat. Struct. Mol. Biol. 27, 450–460. https://doi.org/10.1038/s41594-020-0418-4.
